# Assessments of prostate cancer cell functions highlight differences between a pan‐PI3K/mTOR inhibitor, gedatolisib, and single‐node inhibitors of the PI3K/AKT/mTOR pathway

**DOI:** 10.1002/1878-0261.13703

**Published:** 2024-08-02

**Authors:** Adrish Sen, Salmaan Khan, Stefano Rossetti, Aaron Broege, Ian MacNeil, Ann DeLaForest, Jhomary Molden, Laura Davis, Charles Iversrud, Megan Seibel, Ross Kopher, Stephen Schulz, Lance Laing

**Affiliations:** ^1^ Celcuity, Inc. Minneapolis MN USA

**Keywords:** gedatolisib, inhibitors, PI3K/AKT/mTOR pathway, prostate cancer

## Abstract

Metastatic castration‐resistant prostate cancer (mCRPC) is characterized by loss of androgen receptor (AR) sensitivity and oncogenic activation of the PI3K/AKT/mTOR (PAM) pathway. Loss of the PI3K regulator PTEN is frequent during prostate cancer (PC) initiation, progression, and therapeutic resistance. Co‐targeting the PAM/AR pathways is a promising mCRPC treatment strategy but is hampered by reciprocal negative feedback inhibition or feedback relief. Most PAM inhibitors selectively spare (or weakly inhibit) one or more key nodes of the PAM pathway, potentiating drug resistance depending on the PAM pathway mutation status of patients. We posited that gedatolisib, a uniformly potent inhibitor of all class I PI3K isoforms, as well as mTORC1 and mTORC2, would be more effective than inhibitors targeting single PAM pathway nodes in PC cells. Using a combination of functional and metabolic assays, we evaluated a panel of PC cell lines with different *PTEN*/*PIK3CA* status for their sensitivity to multi‐node PAM inhibitors (PI3K/mTOR: gedatolisib, samotolisib) and single‐node PAM inhibitors (PI3Kα: alpelisib; AKT: capivasertib; mTOR: everolimus). Gedatolisib induced anti‐proliferative and cytotoxic effects with greater potency and efficacy relative to the other PAM inhibitors, independent of *PTEN*/*PIK3CA* status. The superior effects of gedatolisib were likely associated with more effective inhibition of critical PAM‐controlled cell functions, including cell cycle, survival, protein synthesis, oxygen consumption rate, and glycolysis. Our results indicate that potent and simultaneous blockade of all class I PI3K isoforms, mTORC1, and mTORC2 could circumvent PTEN‐dependent resistance. Gedatolisib, as a single agent and in combination with other therapies, reported promising preliminary efficacy and safety in various solid tumor types. Gedatolisib is currently being evaluated in a Phase 1/2 clinical trial in combination with darolutamide in patients with mCRPC previously treated with an AR inhibitor, and in a Phase 3 clinical trial in combination with palbociclib and fulvestrant in patients with HR+/HER2− advanced breast cancer.

## Graphical abstract

Increased activation of the PI3K/AKT/mTOR (PAM) pathway is frequent in prostate cancer (PC), often as a resistance mechanism to androgen therapy. Pan‐PI3K/mTOR inhibitors like gedatolisib inhibit critical PAM‐controlled functions more effectively than single‐node PAM inhibitors, thus achieving greater growth‐inhibitory effects in PC cells, regardless of PTEN status or androgen sensitivity.

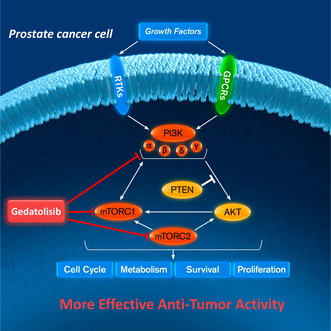

**Abbreviations**
4EBP1: eukaryotic translation initiation factor 4E‐binding protein 1
ADT: androgen deprivation therapy
Alpe: alpelisib
AOC: area over the curve
AR: androgen receptor
BID: twice a day
Capi: capivasertib
CCLE: cancer cell line encyclopedia
ClCasp3: cleaved caspase 3
Copa: copanlisib
CRPC: castration‐resistant prostate cancer
DHT: dihydrotestosterone
DMSO: dimethyl sulfoxide
EdU: 5‐ethynyl‐2′‐deoxyuridine
Eve: everolimus
FBS: fetal bovine serum
FKBP5: FKBP prolyl isomerase 5
FOXO: forkhead box O
FSB: FACS staining buffer
Geda: gedatolisib
GPCR: G protein‐coupled receptor
GR: growth rate
GSEA: gene set enrichment analysis
GSK3: glycogen synthase kinase 3
Ipata: ipatasertib
LPA: 1‐oleoyl lysophosphatidic acid
mCRPC: metastatic castration‐resistant prostate cancer
Mt: mutant
mTOR: mammalian target of rapamycin
mTORC1: mTOR complex 1
mTORC2: mTOR complex 2
OCR: oxygen consumption rate
OPP: *O*‐propargyl‐puromycin
OXPHOS: oxidative phosphorylation
P: phosphorylation
p4EBP1: phosphorylated 4EBP1
PAM: PI3K/AKT/mTOR
PBS: phosphate‐buffered saline
PC: prostate cancer
PDK1: 3‐phosphoinositide‐dependent protein kinase‐1
PHLPP: PH domain and leucine‐rich repeat protein phosphatases
PI3K: phosphatidylinositol 3‐kinase
PIK3CA: PI3K subunit alpha
PIP2: phosphatidylinositol (4,5)‐bisphosphate
PIP3: phosphatidylinositol (3,4,5)‐trisphosphate
pRPS6: phosphorylated RPS6
PTEN: phosphatase and tensin homolog
Q4D: every 4 days
Rheb: Ras homolog, mTORC1 binding
RNA‐seq: RNA sequencing
RPS6: ribosomal protein S6
RTKs: receptor tyrosine kinases
RU: relative units
S6K: S6 kinase
Samo: samotolisib
STR: short tandem repeat
Tase: taselisib
TCA: tricarboxylic acid cycle
TGI: tumor growth inhibition
TME: tumor microenvironment
TSC: tuberous sclerosis complex

## 1 Introduction

Prostate cancer (PC) cell growth typically relies on activation of androgen receptor (AR) signaling by systemic/circulating androgens. At early stages of the disease, androgen deprivation therapy (ADT) and AR signaling inhibitors are effective. However, prostate tumor cells often develop resistance mechanisms that make them less sensitive to AR inhibition and more reliant on alternative pathways, such as the PI3K‐AKT–mTOR (PAM) signaling pathway. As a consequence, new therapies are required to treat PC that progresses to castration‐resistant prostate cancer (CRPC), which has a much poorer prognosis [1, 2].

**Fig. 1:**
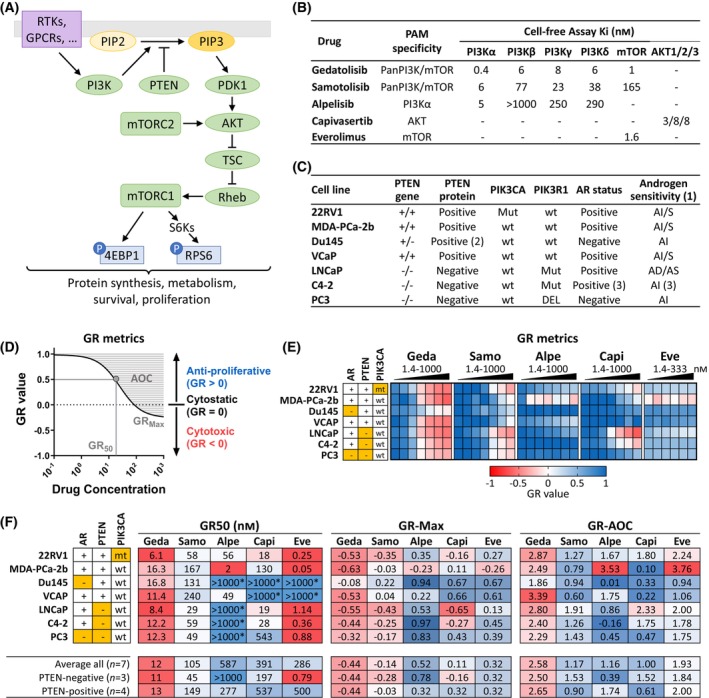
Analysis of PI3K/AKT/mTOR (PAM) inhibitors response in prostate cancer (PC) cell lines using growth rate (GR) metrics. (A) Simplified scheme of the PAM pathway. (B) Gedatolisib, samotolisib, alpelisib, capivasertib, and everolimus target PAM pathway nodes with different specificity and affinity [17, 39, 50, 82, 83]. (C) PC cells lines used in this study. (1) AD , androgen‐dependent (require androgens to grow), AI, androgen independent (do not require androgens to grow), AI/S, androgen independent/androgen responsive (do not require androgens to grow, but show a growth response in their presence) [30]; (2) Heterozygous PTEN; (3) Androgen receptor (AR) expression lower than parental LNCaP [31]; (D) GR metrics are used to assess drugs anti‐proliferative effects (GR value = 0–1), cytotoxic effects (GR < 0), potency (GR_50_), and efficacy (GR_Max_). Efficacy and potency can also be assessed by GR_AOC_ (area over the curve). Lower GR_50_ indicates higher potency; higher negative GR_Max_ indicates higher efficacy; higher GR_AOC_ indicates higher potency and efficacy. (E) Heatmap showing average GR values (*n* = 2) in seven PC cell lines treated for 72 h with increasing PAM inhibitors concentrations. See Table S3 for data. (F) Summary of PAM inhibitors GR_50_, GR_Max_ and GR_AOC_ in the PC cell lines tested. Average values in PTEN‐positive and PTEN‐negative subpopulations are shown. *Max concentration tested, GR_50_ not reached. The results in (E, F) are representative of two separate experiments. Orange cells = AR‐negative, PTEN‐negative, or *PIK3CA*‐mutant (mt). 4EBP1, eukaryotic translation initiation factor 4E‐binding protein 1; alpe, alpelisib; AOC, area over the curve; capi, capivasertib; eve, everolimus; geda, gedatolisib; GPCR, G protein‐coupled receptor; mTORC1, mTOR complex 1; mTORC2, mTOR complex 2; P, phosphorylation; PDK1, 3‐phosphoinositide‐dependent protein kinase‐1; PI3K, phosphatidylinositol 3‐kinase; PIP2, phosphatidylinositol (4,5)‐bisphosphate; PIP3, phosphatidylinositol (3,4,5)‐trisphosphate; Rheb, Ras homolog, mTORC1 binding; RPS6, ribosomal protein S6; RTKs, receptor tyrosine kinases; S6K, S6 kinase; samo, samotolisib; TSC, tuberous sclerosis complex.

The PAM pathway controls many aspects of cell physiology, including cell survival and proliferation, protein synthesis, and metabolic homeostasis. Key nodes of this pathway include class I PI3K enzymes with different catalytic subunit isoforms (p110α, β, γ, δ), AKT, mTOR complex 1 (mTORC1), and mTOR complex 2 (mTORC2) (Fig. 1A). PI3K is activated in response to extracellular signals (e.g., nutrients, hormones, growth factors) through different membrane receptors (e.g., receptor tyrosine kinases, RTKs). Active PI3K converts phosphatidylinositol (4,5)‐bisphosphate (PIP2) into phosphatidylinositol (3,4,5)‐trisphosphate (PIP3), which triggers the activation of a cascade of downstream targets, most notably AKT. One of the main AKT effectors is mTORC1, which promotes anabolic processes necessary for cell growth and proliferation. AKT can also regulate other effectors, such as Forkhead Box O (FOXO) and Glycogen synthase kinase 3 (GSK3), which control many other cellular functions, including cell cycle, cell survival, and cell metabolism. AKT is also phosphorylated and activated by mTORC2, which is another downstream PI3K effector providing additional control over AKT activity. The PTEN phosphatase, by converting PIP3 to PIP2, represents one of main repressing, termination mechanisms of the PAM signaling pathway [3, 4, 5].

Dysregulation of the PAM pathway in PC can be driven by genetic alterations or aberrant expression of PAM pathway genes, such as *PTEN* and *PIK3CA* (encoding the p110α catalytic subunit of PI3K). PTEN loss of function, primarily due to copy‐number loss, has been detected in 15–30% of primary tumors and 40–60% of metastatic CRPC (mCRPC), while *PIK3CA* amplification or activating mutations have been reported in up to 15–30% of CRPC [1, 2, 6, 7]. Animal studies have shown that PTEN loss and PI3K activation have a causal role in prostate tumor growth [8, 9]. Details of reciprocal feedback loops between the androgen signaling and the PAM signaling tie treatment with AR signaling inhibitors indirectly to increased PAM activation. For instance, AR inhibition can activate AKT by reducing the levels of the AKT phosphatase, PHLPP, via FKBP5 chaperone reduction [10].

From a mechanistic standpoint, increased activation of the PAM signaling can drive tumor development and progression by affecting multiple cellular functions, e.g. by promoting cell cycle progression, counteracting pro‐apoptotic signals, and inducing metabolic adaptations required to sustain tumor growth (e.g., increased glycolytic activity) [11]. Since tumor cells heavily rely on PAM‐controlled cellular functions, targeting this pathway is a promising strategy for cancer treatment, and several small molecules inhibiting single or multiple nodes of the PAM pathway have been developed [12]. Several single‐node PAM inhibitors are FDA‐approved for advanced breast cancer in combination with hormonal therapy, including everolimus (mTORC1 inhibitor), alpelisib (PI3Kα inhibitor), and capivasertib (AKT inhibitor). Currently, there are no FDA‐approved PAM inhibitors for PC, but several PAM inhibitors (e.g. samotolisib, everolimus, capivasertib) have been or are currently being evaluated in phase 2/3 PC clinical trials in combination with AR signaling inhibitors [2].

Due to the complexity of the PAM pathway and the many cellular functions controlled by this pathway to preserve cell viability, the efficacy of single‐node PAM inhibitors can be limited by feedback loops and compensatory mechanisms, even when a PAM inhibitor is combined with other therapies [13, 14]. Partly due to these mechanisms, single‐node PAM inhibitors can show different efficacy in cancer cell contexts with different PAM pathway mutations. For instance, AKT inhibitors seem to be more effective in PC cells with PTEN loss, while PIK3α inhibitors seem to be more effective in PC cells with mutated *PIK3CA* and wild‐type *PTEN* [15]. Inhibition of multiple nodes of the PAM pathway by dual PI3K/mTOR inhibitors are expected to overcome *de novo* adaptive resistance mechanisms associated with single‐node antagonisms and, consequently, be effective in a broader patient population [14]. However, while potentially more efficacious, many dual PI3K/mTOR inhibitors cannot be used at effective concentrations due to the route of administration or dose‐limiting toxicities. For instance, the dual PI3K/mTOR inhibitor dactolisib has shown good efficacy in non‐clinical models, but clinical testing in various tumor types, including mCRPC (NCT01717898), has been discontinued due to toxicity [16].

Gedatolisib is a reversible, ATP‐competitive, dual PI3K/mTOR inhibitor targeting all class I PI3K isoforms, as well as mTORC1 and mTORC2, with similar potencies [17, 18]. Early clinical studies showed preliminary gedatolisib efficacy in multiple tumor types with fewer class‐associated adverse effects (e.g. skin and gastrointestinal toxicities, hyperglycemia) relative to published data for other PAM inhibitors [19, 20, 21, 22, 23, 24, 25, 26]. A global Phase 3 clinical trial (VIKTORIA‐1, NCT05501886) is currently evaluating gedatolisib in combination with fulvestrant, with and without palbociclib, in patients with HR+/HER2− advanced breast cancer. In addition, gedatolisib is also being evaluated in combination with darolutamide in patients with mCRPC previously treated with an AR inhibitor in a Phase 1/2 clinical trial (CELC‐G‐201, NCT06190899).

To characterize how PAM pathway node inhibition controls specific PC cell activities, the present study employed multiple functional analyses to compare the effects of gedatolisib to another pan‐PI3K/mTOR inhibitor (samotolisib, weaker mTOR inhibition) and to single‐node PAM inhibitors (alpelisib, capivasertib, everolimus) in PC cell lines with various PTEN status and androgen sensitivity. We demonstrated that equipotent pan‐PI3K/mTOR inhibition by gedatolisib was more effective than the other PAM inhibitors at attenuating multiple PAM‐controlled cellular and metabolic functions driving tumor growth, including protein synthesis, cell cycle progression, cell survival, and cell migration. Consequently, gedatolisib exerted greater anti‐proliferative and cytotoxic effects than the other PAM inhibitors tested, regardless of PTEN status or androgen sensitivity.

## 2 Materials and methods

### 2.1 Cell culture

The following prostate cancer cell lines were obtained from ATCC (Manassas, VA, USA): 22Rv1 (RRID: CVCL_1045), MDA‐PCa‐2b (RRID: CVCL_4748), Du145 (RRID: CVCL_0105), VCaP (RRID: CVCL_2235), LNCaP (RRID: CVCL_0395), LNCaP C4‐2 (RRID: CVCL_4782; here referred to as C4‐2), PC3 (RRID: CVCL_0035). Cell lines were authenticated by STR profiling (ATCC) and tested negative for mycoplasma. Cells were maintained based on ATCC recommendations in growth medium supplemented with 10–20% FBS (R&D Systems, Minneapolis, MN, USA, lot G21162) in a humidified incubator at 37 °C and 5% CO_2_. The same lot of FBS was used throughout the study. According to published data, the amount of testosterone and dihydrotestosterone (DHT) in FBS is ~ 1 ng·mL^−1^ (~ 3.4 nm), which is within the adult men's physiological low range (< 2.4 ng·mL^−1^) [27]. Cell viability experiments were also performed in medium supplemented with charcoal‐stripped FBS (which is depleted of androgens) and 1 nm DHT and showed PAM inhibitor responses similar to the ones observed in medium supplemented with regular FBS (Table S1). Cells were passaged when sub‐confluent and used for experiments at early passages after thawing. The cell lines' PTEN expression, AR expression, and androgen sensitivity were based on previous studies [28, 29, 30, 31]. *PIK3CA* driver alterations were identified by analysis of the Cancer Cell Line Encyclopedia (CCLE, Broad 2019 dataset) through cBioPortal (https://www.cbioportal.org/).

### 2.2 Treatments

Gedatolisib, samotolisib, alpelisib, capivasertib, everolimus, copanlisib, taselisib, and ipatasertib (Selleckchem, Houston, TX, USA) were reconstituted in DMSO and stored in aliquots at −30 °C. Cells were diluted in culture medium and seeded on white 96‐well plates coated with a mixture of fibronectin (Sigma‐Aldrich, St. Louis, MO, USA) and collagen 1 (Advance Biomatrix, Carlsbad, CA, USA) or collagen 1, fibronectin, and laminin‐521 (BioLamina, Sundbyberg, Sweden) in a total volume of 180 μL. Each cell line was seeded at a density optimized to ensure that untreated cells did not reach confluency by the end of the experiment. Cells were let attach overnight and treated with PAM inhibitors or relative vehicle (DMSO) by adding 20 μL of 10× drug freshly diluted in medium as previously described [32].

### 2.3 Cell viability and proliferation‐normalized growth rate assays

After a 72‐h treatment with PAM inhibitors or DMSO, cells were analyzed for cell viability by using the RT‐Glo MT luciferase assay (Promega, Madison, WI, USA) as previously described [32]. The RTGlo MT enzyme and substrate were diluted 1 : 600 in warm medium and 40 μL/well were added to 96‐well plates containing 200 μL medium/well. Plates were incubated for 1 h in a cell culture incubator at 37 °C and 5% CO_2_, and luminescence was measured with an Infinite M1000 (Tecan, Mannedorf, Switzerland) microplate reader. Wells with culture medium + RTGlo MT were used for background subtraction. Endpoint background‐subtracted luminescence was normalized to DMSO‐treated cells (set as 1) to obtain relative viability values. RTGlo MT measurements before and after 72 h PAM inhibitor treatment were used to calculate normalized growth rate (GR) inhibition as described [33]. The GR values at time “*t*” in the presence of drug at concentration “*c*” were calculated using the formula GR(*c*, *t*) = 2*k*(*c*, *t*)/*k*(0) − 1 where *k*(0) is the growth rate of untreated control cells and *k*(*c*, *t*) is the growth rate of drug‐treated cells. GR values between 0 and 1 indicate an anti‐proliferative effect; GR values = 0 indicate a complete cytostatic effect; and GR values between −1 and 0 indicate a cytotoxic effect. GR_50_ (concentration required to obtain a GR value = 0.5) and GR_Max_ (GR value at the maximum concentration tested) were calculated from dose response curves to assess drug potency and efficacy, respectively. The area over the curve (GR_AOC_) was used to assess potency and efficacy at the same time without the constraint of curve fitting [33]. prism (GraphPad Software, Boston, MA, USA) was used to plot DRCs and to calculate the IC_50_ and the GR_50_ of the various PAM inhibitors. GR_Max_ and GR_AOC_ were calculated with the online GR calculator tool (http://www.grcalculator.org/grcalculator/).

### 2.4 CELsignia test

For the CELsignia test, cells were counted using an NucleoCounter NC‐250 (Chemometec, Allerod, Denmark) and seeded in duplicate into 96‐well E‐plates (Agilent, Santa Clara, CA, USA) coated with collagen 1 and fibronectin. Real‐time live cell responses to 1‐oleoyl lysophosphatidic acid (LPA) (Bio‐techne Tocris, Minneapolis, MN, USA) with and without gedatolisib (Selleckchem) were measured and quantified using an xCELLigence RTCA impedance biosensor (Agilent) as described previously [32, 34, 35]. After attachment, cells were treated for 18 h of gedatolisib before addition of 125 nm LPA for 4 h. Impedance changes were recorded throughout the experiment and analyzed using tracedrawer (Ridgeview Instruments AB, Uppsala, Sweden). LPA signal inhibition by gedatolisib was calculated as previously described [34, 35].

### 2.5 Flow cytometry

Flow cytometry analyses were performed on cells collected from 96 well plates after drug treatment for the indicated time as previously described [32]. For DNA replication analysis, cells were incubated with 10 μm 5‐ethynyl‐2′‐deoxyuridine (EdU) (Thermo Fisher, Waltham, MA, USA) for the last 2 h of drug treatment. For protein synthesis analysis, cells were incubated with 5 μm *O*‐propargyl‐puromycin (OPP) (Thermo Fisher) for the last 30 min of drug treatment. At the end of the treatment, both attached cells and floating cells in the culture medium were collected for analysis. Cells were detached with 0.25% Trypsin (Corning, Corning, NY, USA) + 0.5 mm EDTA (Amresco, Solon, OH, USA), blocked with 0.3% Ovomucoid trypsin inhibitor (Worthington, Lakewood, NJ, USA), and transferred to a deep‐well 96 well plate. Cells were spun down at 300 ***g*** for 7 min at 4 °C, washed with PBS, stained with Zombie NIR viability dye (Biolegend, San Diego, CA, USA) for 15 min at room temperature, washed with FACS staining buffer (FSB: PBS + 0.5% BSA + 0.02% Sodium Azide), fixed with 1.6% paraformaldehyde for 10 min (Electron Microscopy Sciences, Hatfield, PA, USA), and permeabilized with cold ACS grade methanol (Sigma) for 15 min. The cells were then used for the following assays.

#### 2.5.1 Analysis of DNA replication, cell cycle, cell death, and apoptosis

DNA replication was assessed by detection of EdU incorporation into newly synthesized DNA, while the cell cycle phases were quantified by DNA staining with FxCycle violet (Thermo Fisher) as previously described [32]. EdU incorporation was detected in fixed and permeabilized cells by using the Click‐iT EdU Alexa Fluor 647 kit (Thermo Fisher) according to the vendor's instructions. After the Click‐iT reaction, cells were washed with FSB buffer, stained with anti‐cleaved caspase 3‐A488 antibody (Cell Signaling, Danvers, MA, USA; 1 : 20 dilution) for 30 min at 4 °C, washed with FSB, and run on a Novocyte 3005 (Agilent) flow cytometer. NovoExpress 1.5.6 (Agilent) was used for gating and data analysis. Dead cells were identified by Zombie staining, and live apoptotic cells were identified by cleaved caspase 3 staining within the Zombie‐negative (live) cells. The live cell population was further gated for EdU incorporation to identify cells with active DNA replication, and FxCycle Violet to identify G0/G1, S, and G2/M phases. EdU incorporation data were normalized to DMSO‐treated control cells (set at 1). EdU incorporation and cell death were also analyzed using a staining panel containing Zombie NIR, EdU‐A647, anti‐pRPS6‐BV421(S235/S236) (Biolegend; 1 : 50 dilution) and anti‐p4EBP1‐AF488 (T36/T45) (BD Biosciences, Franklin Lakes, NJ, USA; 1 : 25 dilution).

#### 2.5.2 Analysis of PAM pathway activity and protein synthesis

PAM pathway activity was assessed by detection of p4EBP1(T36/T45) and pRPS6‐BV421(S235/S236), while protein synthesis was assessed by quantification of OPP incorporation into newly synthesized proteins as previously described [32]. After fixation and permeabilization, cells were stained for OPP incorporation with Click‐iT OPP Alexa Fluor 647 kit (Thermo Fisher) based on the vendor's instructions. Following the Click‐iT reaction, cells were washed with FSB buffer, stained with anti‐pRPS6‐BV421(S235/S236) antibody (Biolegend; 1 : 25 dilution) and anti‐p4EBP1‐AF488 (T36/T45) antibody (BD Biosciences; 1 : 25 dilution) and run on a Novocyte 3005 (Agilent) flow cytometer. OPP incorporation and pRPS6 and p4EBP1 median levels were quantified in live cells gated by Zombie staining and normalized to DMSO‐treated control cells (set at 1).

### 2.6 Metabolic studies

The oxygen consumption rate (OCR) was measured on a Resipher instrument (Lucid Scientific, Inc., Atlanta, GA, USA) as previously described [32]. Cells were seeded in a 96‐well plate coated with collagen 1/fibronectin or collagen 1/fibronectin/laminin‐521in 180 μL/well of growth medium and let attach for 24 h. After attachment, cells were treated by adding 20 μL/well of a 10× drug freshly diluted in medium. Control cells were treated with an equivalent amount of DMSO. Oxygen levels were monitored on the Resipher during a 24 h treatment. At the end of the treatment the live cell number was quantified by flow cytometry using Sytox blue stain (Thermo Fischer) to exclude dead cells. Where indicated, the OCR was normalized to the number of live cells at the end of the experiment.

Glucose and lactate levels were measured using the Biosen R‐line instrument (EKF Diagnostic, Cardiff, UK) as previously described [32]. Cells were seeded in a coated 96‐well plate in 100 μL/well of growth medium, let attach for 24 h, and treated by adding 10 μL/well of an 11× drug freshly diluted in medium. After 24 h of treatment, 10 μL of conditioned medium was added to 500 μL glucose/lactate hemolyzing solution (EKF Diagnostics), mixed, and analyzed on the Biosen R‐line instrument. Lactate production was calculated by subtracting the baseline level of lactate present in non‐conditioned medium from the lactate level in the conditioned medium. Glucose consumption was calculated by subtracting the glucose level in the conditioned medium from baseline glucose level in non‐conditioned medium. The cell number at the end of treatment was assessed by flow cytometry as described above and used to normalize lactate production and glucose consumption.

Glucose uptake was assessed by Glucose Uptake‐Glo assay (Promega) according to the vendor's protocol. Cells were seeded on coated 96‐well plates. After 16 h incubation, cells were treated with PAM inhibitors for 4 h. Cells were then washed twice with PBS and incubated with 1 mm 2‐deoxyglucose for 10 min. Detection reagent was applied for 1 h to detect 2‐deoxyglucose‐6‐phosphate in the cells with an Infinite M1000 (Tecan) microplate reader. RT‐Glo MT luciferase assay was used to normalize glucose uptake to viable cells.

### 2.7 Migration studies

Cell migration was determined using a transwell assay with permeable support inserts for 24‐multiwell plate with 8 μm pores (Corning) as previously described [32]. 7.5 × 10^4^ Du145 cells resuspended in 0.5 mL FBS‐free growth medium were added to the permeable inserts (upper chamber of the transwell assay). Adjacent wells (lower chamber of the transwell assay) contained 0.75 mL growth media supplemented with 10% FBS and 150 nm 1‐Oleoyl lysophosphatidic acid sodium salt (LPA; Bio‐techne Tocris) as migration stimulus/attractant. Drugs at indicated doses were added to both upper and lower chambers. Two hours after cells were seeded, the upper chamber was moved to the adjacent wells containing the FBS/LPA‐supplemented medium. The cells were then allowed to migrate for approximately 24 h. The bottom of the insert containing the migrated cells was washed, fixed, and stained with a 1% crystal violet aqueous solution (Sigma‐Aldrich). The migrated cells were imaged, and the stain was eluted with 33% acetic acid solution for quantitation using Infinite M1000 (Tecan) microplate reader at an absorbance of 570 nm.

### 2.8 *In vivo* efficacy studies

The *in vivo* studies were performed at Crown Biosciences (Zhongshan, China) in adherence with Crown Biosciences Animal welfare guidelines and following approved Institutional Animal Care and Use Committee (IACUC) protocols. The *in vivo* studies were approved by Crown Biosciences IACUC under license number SOP‐G‐006(CBCN). Male BALB\c nude mice at 6–8 weeks of age were ordered from GEMPharmatech Co. Ltd (Nanjing, China). On arrival at Crown Biosciences, mice were transferred into polysulfone IVC disposable cages and stored on HEPA ventilated Innoracks in a temperature and humidity‐controlled housing room. Animals were maintained on a 12‐h light–dark cycle (lights on at 6 am and lights off at 6 pm) with access to irradiated food and softened autoclaved water *ad libitum*. Following delivery and acclimatization, mice were anesthetized with ketamine/xylazine and surgically castrated. After exposing each testicle via a midline scrotal incision, the spermatic cord was ligated with a 6‐0 Vicryl suture, and the testicle was removed; the scrota and skin were then closed with 6‐0 Vicryl suture, separately. Three days after castration, each mouse was inoculated subcutaneously in the right upper region with 22Rv1 (1 × 10^7^) or PC3 (5 × 10^6^) tumor cells resuspended in 100 μL of 50% PBS/50% Matrigel. When the mean tumor size reached 120–130 mm^3^, mice were randomized into two arms of 10 mice each (vehicle and gedatolisib) based on the “Matched distribution” method (study director™ software, version 3.1.399.19). The date of grouping was denoted as day 0. Clinically formulated gedatolisib (Celcuity, Minneapolis, MN, USA) was resuspended in water and administered intravenously Q4D for 28 days. Mice were weighed daily and monitored for signs of morbidity. Tumor volumes were measured in two dimensions twice per week using a caliper. The tumor volume, expressed in mm^3^, was calculated using the formula: *V* = (*L* × *W* × *W*)/2, where *V* is the tumor volume, *L* is the tumor length (longest dimension) and *W* is the tumor width (longest dimension perpendicular to *L*). Tumor growth inhibition (TGI) was determined by the formula: %TGI = [1 − (*V*_tx_ − *V*_t0_/*V*_cx_ − *V*_c0_)] × 100, where *V*_c_, *V*_t_ are the means of control and treated groups, respectively, x is the day on study (28 days) and 0 is the day of first dosing. Statistical significance was calculated by one‐way ANOVA.

### 2.9 Pharmacokinetic studies

Pharmacokinetic analysis of gedatolisib concentration in xenograft tumor tissue was performed by OpAns, LLC (Durham, NC, USA). Tumor tissue samples collected at various time points after gedatolisib treatment were frozen and analyzed by HPLC/MS–MS. Chromatographic separations were performed using a 1290 series high pressure liquid chromatography (HPLC) system (Agilent Technologies) with a 3 × 50 mm reversed phase column at 40 °C. Mass spectrometric analysis was performed using an Agilent 6495 Series Triple Quadrupole tandem mass spectrometer equipped with an Agilent Jet Stream Electrospray source using nitrogen as the carrier gas and operated in the positive ion mode. HPLC/MS–MS data acquisition and processing were performed using MassHunter Workstation Data Acquisition for Triple Quad (Agilent).

### 2.10 Bioinformatic analyses

The Prostate Cancer Transcriptome Atlas includes sequencing data from 1223 clinical samples and was used to infer the locations of pathway‐specific transcripts on a pseudotime disease progression trajectory as described in Bolis et al. [36] using the tidyverse, dplyr, and ggplot R packages. Pathway‐specific transcripts were curated from GSEA hallmark gene signatures for PI3K/mTOR (overlap genes between the *HALLMARK_PI3K_AKT_MTOR_SIGNALING* and the *HALLMARK_MTORC1_SIGNALING* gene sets) and glucose metabolism (*REACTOME: glucose metabolism human gene* set). A transcriptional signature for hypoxia was created by merging a previously described 15‐gene universal hypoxia classifier [37] and a PCa‐specific hypoxia signature [38]. In Bolis et al. [36], a prostate cancer patient RNA‐seq dataset (*N* = 1106 subjects) is analyzed to assign to each patient a unique pseudotime value (ranging from 0 to 250), reflecting the patient's position upon a clinical disease progression timeline based on the patient's overall genome expression state. In addition, Bolis et al. derive a Pearsons's Correlation Coefficient (PCC), computing the correlation between pseudotime (i.e., disease progression) and mRNA expression increase or decrease for each human gene into a single value that ranges between −1 and +1. Thus, *X*‐axis negative values between −1 and zero represent gene expression that starts at a higher level in the early stages of disease clinical presentation and decreases as the disease progresses. *X*‐axis positive values between zero and +1 correspond to gene expression increases as patients with PC progress to metastatic and neuroendocrine clinical presentations of disease. Using this data framework, different mRNAs (or transcriptional signatures) can be visualized on a clinical PC disease progression trajectory. In the temporal transcript‐mapping plots shown in our study, the *X*‐axis displays the Pearsons's Correlation Coefficient (PCC) for the transcriptional signatures analyzed. False discovery rate *q*‐values of mRNA‐pseudotime associations are plotted on the *Y*‐axis as negative log_10_ FDR; higher *Y*‐axis values correspond to more robust associations with disease progression in either a positive or negative RNA expression change direction. Cutoffs of FDR *q*‐value < 0.05 and < 0.00005 correspond to *Y*‐axis values of PCC ~ 13 and ~ 43, respectively, on the negative logarithmic scale.

### 2.11 Statistical analyses

Statistical significance was calculated as indicated in the figure legends using prism (GraphPad). *P*‐values < 0.05 were considered significant.

## 3 Results

### 3.1 Analysis of PC cell lines response to PAM inhibitors by cell viability assay and growth rate metrics

To compare the growth‐inhibitory effects of multi‐node versus single‐node PAM inhibitors, seven PC cell lines were tested for cell viability in response to treatment with gedatolisib, samotolisib (pan‐PI3K/mTOR inhibitors), capivasertib (AKT inhibitor), alpelisib (PI3Kα inhibitor), or everolimus (mTORC1 inhibitor) (Fig. 1B). While gedatolisib and samotolisib have both been described as pan‐PI3K/mTOR inhibitors [17, 18, 39], they differ in potency and uniformity of inhibition. Gedatolisib shows equi‐nanomolar cell‐free IC_50_ against all class I PI3K isoforms and mTOR (0.4–8 nm), while samotolisib cell‐free IC_50_ for mTOR (165 nm) is > 25‐fold higher than the IC_50_ for PI3Kα (6 nm); additionally, samotolisib is also > 10‐fold less potent against PI3Kβ (IC_50_ 75 nm) than PI3Kα (Fig. 1B). In order to evaluate the impact of PAM alterations or androgen sensitivity on PAM antagonism, the cell lines were chosen to represent subpopulations with different *PIK3CA*, PTEN, and AR status (Fig. 1C).

Cells treated with increasing concentrations of each PAM inhibitor for 72 h were analyzed by classical endpoint cell viability metrics (shown in Fig. S1 and Table S2) as well as by growth rate (GR) metrics, with similar results. Differentiated from endpoint cell viability analyses, the GR metrics approach can identify both anti‐proliferative and cytotoxic drug effects without confounding effects due to the different division rates of the cell lines tested (Fig. 1D) [33]. The GR metrics analysis indicated that gedatolisib induced anti‐proliferative and cytotoxic effects in a dose‐dependent fashion (Fig. 1E and Table S3). Gedatolisib GR_50_ (a measure of potency) ranged from 6 to 17 nm and the GR_Max_ (a measure of efficacy) ranged from −0.1 to −0.6, indicating potent cytotoxic effects in all cell lines tested, regardless of *PIK3CA* status, PTEN expression, AR expression, or androgen sensitivity (Fig. 1F).

On average, gedatolisib generally showed greater potency and efficacy than the other PAM inhibitors, in both PTEN‐negative and PTEN‐positive cell lines. In PTEN‐negative cells, capivasertib was less potent than gedatolisib, with an average GR_50_ almost 18‐fold higher than gedatolisib GR_50_ (197 nm versus 11 nm, respectively), while alpelisib failed to reach the GR_50_ at concentrations up to 1000 nm. Everolimus showed higher potency (average GR_50_ = 0.8 nm) than gedatolisib in this cell subpopulation but was less potent than gedatolisib in PTEN‐positive cell lines (average GR_50_ = 500 nm versus 13 nm, respectively). Similarly, capivasertib, alpelisib, and everolimus GR_Max_ values were, on average, lower than gedatolisib GR_Max_ (−0.16, 0.78, 0.32 versus −0.44, respectively), indicating lower efficacy. The other pan‐PI3K/mTOR inhibitor, samotolisib, showed greater efficacy than thesingle‐node PAM inhibitors but did not reach the same level of cytotoxicity induced by gedatolisib (average GR_Max_ = −0.28). In PTEN‐positive cells, gedatolisib was more potent and efficacious relative to all the other PAM inhibitors evaluated.

Further analysis of the GR_AOC_, a GR metric capturing both potency and efficacy, where a higher number indicates higher activity, confirmed that gedatolisib exerted greater growth‐inhibitory effects relative to the other PAM inhibitors (average GR_AOC_ = 2.50 for gedatolisib versus GR_AOC_ = 1.53, 0.39, 1.52, and 1.84 for samotolisib, alpelisib, capivasertib, and everolimus, respectively, in PTEN‐negative cells) (Fig. 1F). Of note, gedatolisib displayed similar potency and efficacy in PTEN‐positive and PTEN‐negative cells, while capivasertib and samotolisib were more potent and efficacious in PTEN‐negative cells and alpelisib was more potent and efficacious in PTEN+ cells (see average GR_AOC_ values in Fig. 1F). Additional experiments showed that gedatolisib was also more potent and efficacious than ipatasertib (AKT inhibitor), copanlisib (pan‐PI3K inhibitor with mTOR IC_50_ = 45 nm), and taselisib (PI3Kα,γ,δ inhibitor) (Fig. S2 and Table S3).

In order to detail the functional aspects of gedatolisib's greater potency and efficacy, we set out to investigate the effect of the various PAM inhibitors on PAM‐controlled cancer cell functions known to play a critical role in cancer cell survival and proliferation.

### 3.2 Effects of PAM inhibitors on cell cycle and DNA replication functions

**Fig. 2:**
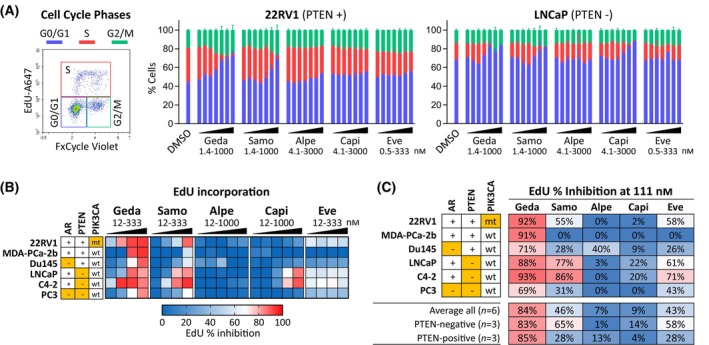
Effects of PI3K/AKT/mTOR (PAM) inhibitors on cell cycle and DNA replication. (A) Flow cytometric analysis of the cell cycle phases by EdU incorporation and FxCycle staining in 22RV1 and LNCaP cell lines treated with PAM inhibitors for 48 h. Data represent mean ± SD (*n* = 2). See Table S4 for data. (B) Heatmap showing average inhibition of EdU incorporation (*n* = 2) in six PC cell lines treated with PAM inhibitors at increasing concentrations for 48 h. See Table S5 for data. (C) Comparison of PAM inhibitors efficacy in inhibiting EdU incorporation at 111 nm. The results are representative of three separate experiments. Orange cells = AR‐negative, PTEN‐negative, or *PIK3CA*‐mutant (mt). A647, Alexa Fluor 647; alpe, alpelisib; capi, capivasertib; EdU, 5‐ethynyl‐2′‐deoxyuridine; eve, everolimus; geda, gedatolisib; samo, samotolisib.

The role of the PAM pathway in promoting cell cycle progression and cell proliferation is well established [3, 4]. To compare the effects of single‐node and multi‐node PAM inhibitors on cell cycle and DNA replication, PC cell lines were exposed to increasing doses of each PAM inhibitor for 48 h and analyzed by flow cytometry for DNA replication and cell cycle distribution by EdU incorporation and DNA staining with FxCycle, respectively (Fig. 2A). Gedatolisib inhibited cell cycle progression in a dose‐dependent manner in both 22RV1 (PTEN+) and LNCaP (PTEN−) cells. Cell cycling was mostly inhibited at the G0/G1 and S phases. Starting from 12 to 37 nm, gedatolisib increased the G0/G1 phase from 44% up to 73% in 22RV1 and from 68% up to 84% in LNCaP at the maximum concentration tested (1000 nm). Concomitantly, DNA replication during the S phase decreased from 37% to 2% in 22RV1 and from 18% to 0% in LNCaP (Fig. 2A and Table S4). Capivasertib effectively reduced the S phase (from 18% to 1%) and increased the G0/G1 phase (from 68% to 88%) at concentrations > 111 nm in LNCaP cells (PTEN−) but had almost no effect in 22RV1 cells (PTEN+). Everolimus and alpelisib slightly decreased the S phase (from 37% to 21–29%) and increased the G0/G1 phase (from 44% to 53–56%) in 22RV1 cells but had no effect on LNCaP. The other pan‐PI3K/mTOR inhibitor, samotolisib, reduced S phase and increased G0/G1 phase to the same degree as gedatolisib in both LNCaP and 22RV1, but at higher concentrations (> 111–333 nm) (Fig. 2A and Table S4).

DNA replication was further analyzed in a panel of 6 PC cell lines with or without PTEN loss after treatment with increasing doses of different PAM inhibitors for 48 h. At the maximum concentration tested, gedatolisib induced > 80% average inhibition of EdU incorporation in all cell lines, while > 80% inhibition was achieved only in three cell lines for samotolisib, two cell lines for capivasertib, and no cell lines for everolimus and alpelisib (Fig. 2B and Table S5). On average, at 111 nm, gedatolisib inhibited EdU incorporation by 84%, compared to 46%, 7%, 9%, and 43% for samotolisib, alpelisib, capivasertib, and everolimus, respectively. Gedatolisib was equally effective in PTEN− and PTEN+ cell lines (83% versus 85% inhibition), while everolimus and samotolisib showed better efficacy in PTEN− cell lines (58–65% inhibition) relative to PTEN+ cells (28% inhibition) (Fig. 2C).

Based on these observations, we concluded that gedatolisib inhibited cell cycle progression and DNA replication more effectively relative to the other PAM inhibitors evaluated, both in PTEN− and PTEN+ PC cell lines.

### 3.3 Effects of PAM inhibitors on cell death and apoptosis functions

**Fig. 3:**
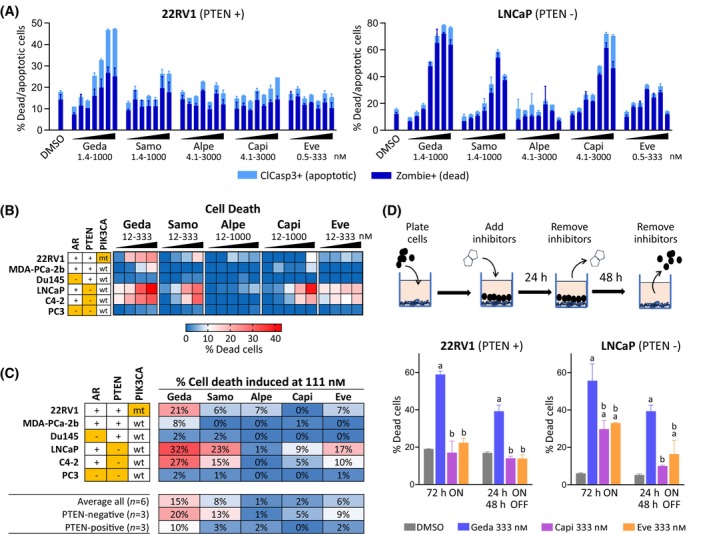
Effects of PI3K/AKT/mTOR (PAM) inhibitors on cell death and apoptosis. (A) Flow cytometric analysis of cell death (Zombie staining) and apoptosis (cleaved caspase 3) in 22RV1 and LNCaP cell lines treated with PAM inhibitors for 48 h. Data represent mean ± SD (*n* = 2). See Table S6 for data. (B) Heatmap showing induction of death by Zombie staining in six PC cell lines treated with PAM inhibitors at increasing concentrations for 48 h. Data represent % dead cells (Zombie+) over DMSO‐treated cells (average of 2 biological replicates). See Table S7 for data. (C) Comparison of PAM inhibitors efficacy in inducing cell death at 111 nm. The results in A–C are representative of three separate experiments. (D) Analysis of cell death (Zombie staining) in PC cell lines treated with PAM inhibitors for either 72 h or 24 h followed by washout and incubation with drug‐free medium for 48 h. Data represent mean ± SD (*n* = 2); a = *P* < 0.05 vs DMSO, b = *P* < 0.05 vs gedatolisib; one‐way ANOVA Fisher's test. Orange cells = AR‐negative, PTEN‐negative, or *PIK3CA*‐mutant (mt). alpe, alpelisib; capi, capivasertib; clCasp3, cleaved caspase 3; eve, everolimus; geda, gedatolisib; samo, samotolisib.

Activation of the PAM pathway is known to promote cell survival through inhibition of pro‐apoptotic pathways [3, 4]. To determine if single‐node and multi‐node PAM inhibitors had different effects on apoptosis and cell death, PC cell lines were treated with increasing concentrations of each PAM inhibitor for 48 h and analyzed by flow cytometry for the presence of dead cells (identified by Zombie staining) or live apoptotic cells (identified by cleaved caspase 3). Gedatolisib induced apoptosis and cell death in a dose‐dependent fashion in both 22RV1 (PTEN+) and LNCaP (PTEN−) cells. Starting from 37 nm, gedatolisib increased the % of dead and apoptotic cells up to 47% in 22RV1 and up to 77% in LNCaP at the maximum concentration tested (1000 nm). Samotolisib, capivasertib and everolimus increased cell death and apoptosis up to 59%, 72%, and 34%, respectively, in LNCaP cells but had very modest effects in 22RV1 cells (PTEN+). Alpelisib did not induced relevant cell death or apoptosis in either 22RV1 or LNCaP cells (Fig. 3A and Table S6).

The effect of gedatolisib and the other PAM inhibitors on cell death was then analyzed in six PC cell lines with different PTEN status (Fig. 3B and Table S7). Treatment with 111 nm gedatolisib for 48 h induced > 20% cell death over DMSO‐treated cells in three of the six cell lines tested (22RV1, LNCaP, C4‐2), compared to one cell line (LNCaP, PTEN−) for samotolisib and no cell lines for alpelisib, capivasertib, and everolimus at the same concentration (Fig. 3C).

Targeting of AKT or mTOR has been suggested to be less effective than PI3K inhibition at inducing rapid (< 24 h) apoptosis and cell death in cancer cells [40]. To compare the effects of a short, transient exposure to PAM inhibitors on cell death, 22RV1 (PTEN+) and LNCaP (PTEN−) cells were treated with gedatolisib or single‐node PAM inhibitors for 24 h followed by a washout and media change with drug‐free medium for 48 h. As a control, cells were also treated continuously with gedatolisib for 72 h. As shown in Fig. 3D, both transient and continuous gedatolisib treatment (333 nm) induced significant cell death in the cell lines tested. Capivasertib and everolimus induce significant LNCaP cell death after continuous treatment for 72 h, but the induction of cell death was greatly reduced when cells were treated for only 24 h (Fig. 3D).

Overall, these results showed that pan‐PI3K/mTOR inhibition, even when transient, induced greater cell death than inhibition of single PAM pathway nodes.

### 3.4 Effects of PAM inhibitors on PAM pathway activity and protein synthesis functions

PAM pathway activation, through mTORC1 and its downstream effectors S6K and 4EBP1, leads to increased protein synthesis [41]. Since cancer cells depend on protein synthesis to maintain their increased growth rate [41, 42], we tested the effect of the different PAM inhibitors on PAM pathway activity and protein synthesis.

**Fig. 4:**
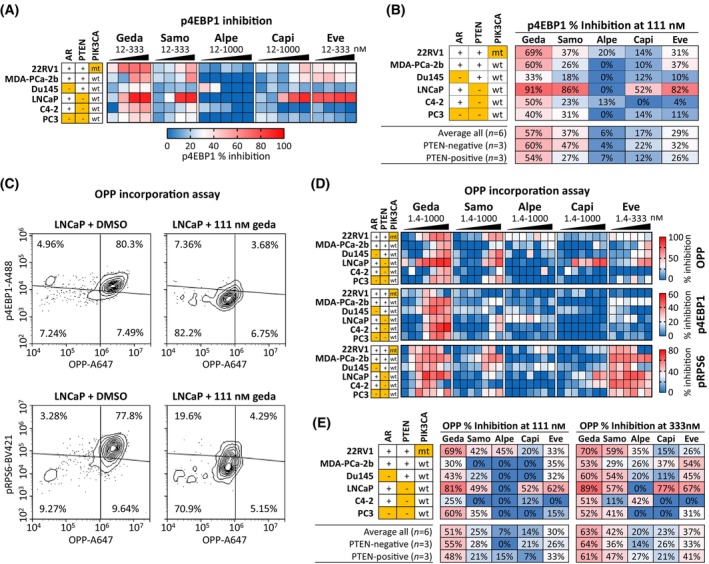
Effects of PI3K/AKT/mTOR (PAM) inhibitors on PAM pathway activity and protein synthesis. (A) Heatmap showing average inhibition (*n* = 2) of p4EBP1 level after 48 h treatment with increasing PAM inhibitors concentrations in six prostate cancer (PC) cell lines. The % inhibition was calculated from median fluorescence and is relative to DMSO‐treated cells. See Table S8 for data. (B) Comparison of PAM inhibitors efficacy in reducing p4EBP1 levels at 111 nm concentration. The results in A and B are representative of one out of three separate experiments. (C) Example of multiplex flow cytometric analysis of OPP incorporation, p4EBP1, and pRPS6 in LNCaP cells treated with 111 nm gedatolisib or DMSO control for 24 h. (D) Heatmap showing average inhibition (*n* = 2) of OPP incorporation, p4EBP1, and pRPS6 in six PC cell lines treated with increasing PAM inhibitors concentrations for 24 h. The % inhibition was calculated from median fluorescence intensity and is relative to DMSO‐treated cells. The values shown in the heatmap represent the average of 2 biological replicates. See Table S9 for data. (E) Comparison of PAM inhibitors efficacy in inhibiting OPP incorporation at 111 nm. Orange cells = AR‐negative, PTEN‐negative, or *PIK3CA*‐mutant (mt). A 647, Alexa Fluor 647; A488, Alexa Fluor 488; Alpe, alpelisib; BV421, Brilliant Violet 421; capi, capivasertib; eve, everolimus; geda, gedatolisib; OPP, *O*‐propargyl‐puromycin; samo, samotolisib.

We first evaluated the effects of the PAM inhibitors on PAM pathway activity by measuring the functional status of p4EBP1(T36/T45), which integrates PAM signaling pathway outputs from both PI3K/mTORC1 and mTORC2/pAKT (Fig. 1A). The six PC cell lines previously used for cell cycle and DNA replication analyses were treated for 48 h with gedatolisib, samotolisib, alpelisib, capivasertib, or everolimus at increasing concentrations, and analyzed for p4EBP1 levels by flow cytometry (Fig. 4A and Table S8). On average, 111 nm gedatolisib inhibited p4EBP1 by 60% in PTEN‐negative cells and 54% in PTEN‐positive cells. At the same concentration, samotolisib, alpelisib, capivasertib, or everolimus inhibited p4EBP1 by 47%, 4%, 22%, and 32% in PTEN‐negative cells and by 27%, 7%, 12%, and 26% in PTEN‐positive cells, respectively (Fig. 4B).

Next, we used the same PC cell lines to compare the PAM inhibitors effects directly on protein synthesis. Cells were treated with each PAM inhibitor for 24 h and incubated with OPP for the last 30 min. OPP is an analog of puromycin that is incorporated into nascent proteins and thus provides an objective quantification of translation. After OPP incubation, cells were analyzed by flow cytometry for OPP, p4EBP1, and pRPS6 (used as indicator of S6Ks activity). As shown for the LNCaP cell line as an example, 111 nm gedatolisib reduced the percentage of pRPS6+/OPP+ cells and p4EBP1+/OPP+ cells from approximately 80% to < 5% (Fig. 4C). Gedatolisib concomitantly reduced OPP incorporation, pRPS6, and p4EBP1 in all cell lines tested (Fig. 4D and Table S9). On average, gedatolisib inhibited OPP incorporation by 51% at 111 nm, and 63% at 333 nm, with little difference between PTEN‐positive and PTEN‐negative cell lines (Fig. 4E). Samotolisib, alpelisib, capivasertib, and everolimus inhibited OPP incorporation ≤ 30% at 111 nm and ≤ 42% at 333 nm (Fig. 4E).

These results showed that gedatolisib inhibited PAM pathway activity and PAM‐controlled protein synthesis more efficaciously than the other PAM inhibitors, regardless of PTEN status.

### 3.5 Effects of PAM inhibitors on glucose, lactate, and O_2_ metabolic functions

Increased glycolysis (with consequent lactate production) and oxidative phosphorylation, which often characterize PC progression, can be exploited by cancer cells to sustain their increased demand for energy production and biosynthesis [43] as well as to modify the tumor microenvironment [44] to patient detriment. Further review of the literature also reported the PAM pathway involvement in driving cancer regardless of PAM pathway gene mutations [45].

**Fig. 5:**
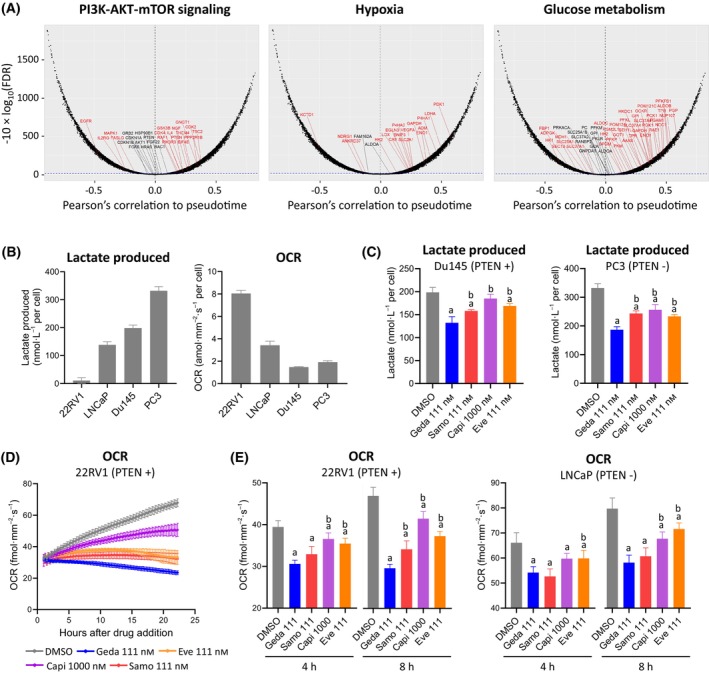
Effects of PI3K/AKT/mTOR (PAM) PAM inhibitors on metabolic functions. (A) Temporal transcript‐mapping plots representing the correlation between mRNA expression and prostate cancer (PC) clinical disease stage. *X*‐axis: Pearson's correlation coefficient (PCC) between pseudotime and mRNA expression; *Y*‐axis: the associated significance between mRNA expression and PC disease progression, adjusted for false discovery rate (FDR), and expressed in the form of −10 × log_10_(FDR) [36]. Transcripts in red are significantly correlated with disease progression (FDR *q*‐value < 0.05). The dashed blue line indicates the cutoff for significance (see Section 2 for details and Table S10 for gene lists). (B) Analysis of lactate levels in conditioned culture medium (24 h culture) and oxygen consumption rates (OCR, 24 h culture) showing that PC cell lines are characterized by different metabolic states. (C) Analysis of lactate levels in conditioned medium of Du145 and PC3 cells treated with PAM inhibitors for 24 h. See Table S11 for data. (D) Example of real‐time OCR Resipher analysis in 22RV1 cells treated with PAM inhibitors for 24 h. Data represent mean ± SD (*n* = 3). (E) OCR analysis in 22RV1 and LNCaP cells treated with PAM inhibitors for 4–8 h. Data in B, C and E represent mean ± SD (*n* = 3); a = *P* < 0.05 vs DMSO, b = *P* < 0.05 vs 111 nm gedatolisib; one‐way ANOVA Fisher's test. See Table S13 for data. Alpe, alpelisib; capi, capivasertib; eve, everolimus; geda, gedatolisib; samo, samotolisib.

In support of these observations, we examined whether alterations of these metabolic features in patients with PC correlated with the status of PAM pathway activity in patients using trajectory inference analyses of the Prostate Cancer Transcriptional Atlas, which combines transcriptional profiles from more than 1000 clinical PC samples to allow mapping specific gene expression modules onto the disease progression trajectory [36]. For patients with advanced disease, pseudotime nomination of genes characteristic of PAM, hypoxia, and glucose metabolism status (from curated gene expression modules for PI3K and mTOR, hypoxia, and glucose metabolism) revealed a shared impact of multiple metabolic functions linked to PAM hyperactivation driving PC disease trajectory (Fig. 5A and Table S10). Since the PAM pathway plays a key role in controlling metabolic activities [11, 46], we hypothesized that inhibition of the PAM pathway at different nodes may have different effects on metabolic functions such as glycolysis and oxygen consumption.

We first assessed the baseline levels of glycolysis and oxygen consumption rate (OCR) in four PC cell lines. Glycolysis was inferred from the production of lactate (the endpoint product of glycolysis) in the conditioned medium of cells cultured from 24 h, while OCR was assessed by real time monitoring of the O_2_ levels during culture using a Resipher instrument. After normalization to cell number, the four PC cell lines displayed a range of metabolic states, with 22RV1 and LNCaP having the highest OCR and lowest lactate production, and Du145 and PC3 having the lowest OCR and the highest lactate production (Fig. 5B).

We next tested the effect of the different PAM inhibitors on OCR and lactate production. To test the PAM inhibitors' effects on glycolysis, we selected the two cell lines with the highest lactate production, Du145 (PTEN+) and PC3 (PTEN−). After 24‐h treatment, gedatolisib inhibited lactate production significantly (*P* < 0.05) more than samotolisib, capivasertib, and everolimus in both Du145 (33% versus 20%, 7%, and 15%, respectively) and PC3 (44% versus 27%, 23%, and 30%, respectively) (Fig. 5C and Table S11). Additional experiments showed that inhibition of lactate production by PAM inhibitors was also associated with a significant reduction in glucose uptake (Fig. S3 and Table S12). Inhibition at AKT and mTOR appears to be generally less effective in controlling glucose metabolism in these cells.

To test the PAM inhibitors' effects on OCR, we selected the two cell lines with the highest baseline OCR, 22RV1 (PTEN+) and LNCaP (PTEN−). Cells were treated with each PAM inhibitor for approximately 24 h, and OCR was monitored throughout the treatment as shown for 22RV1 in Fig. 5D. Since treatment with gedatolisib in these cell lines can induce cell death after 24 h (see Fig. 3D), the PAM inhibitors effect on OCR was measured at earlier time points (4–8 h). After 8 h treatment, gedatolisib inhibited OCR significantly more than samotolisib, capivasertib, and everolimus in 22RV1 (37% versus 27%, 12%, and 21%; *P* < 0.05) and significantly more than capivasertib and everolimus in LNCaP (27% versus 15%, and 10%; *P* < 0.05) (Fig. 5E and Table S13).

These studies showed that equipotent inhibition of multiple nodes of the PAM pathway by gedatolisib targeted key metabolic adaptations of PC cancer cells more effectively than inhibition of single nodes of the PAM pathway.

### 3.6 Effects of PAM inhibitors on cell migration function

**Fig. 6:**
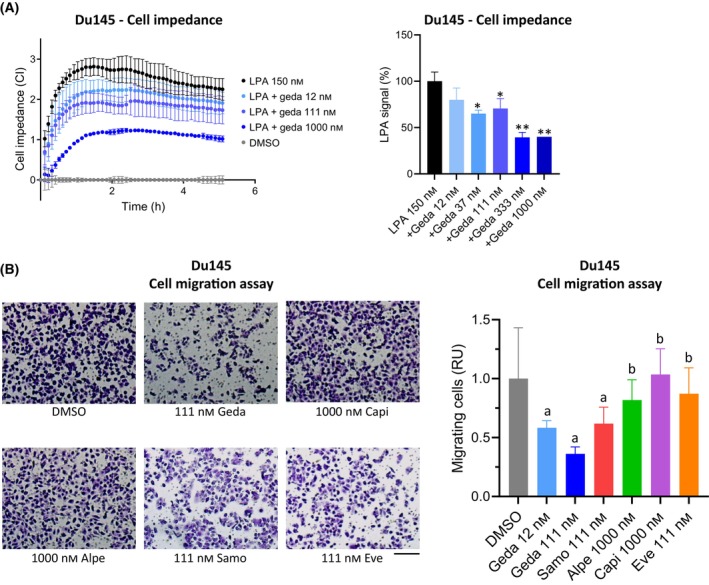
Effects of PI3K/AKT/mTOR (PAM) inhibitors on cell migration. (A) xCELLigence analysis showing that lysophosphatidic acid (LPA)‐induced impedance is inhibited by gedatolisib in Du145 cells. Data represent the mean ± SD (*n* = 2). *P < 0.05, **P < 0.01 versus LPA (no gedatolisib). (B) Transwell assay showing inhibition of cell migration in response to PAM inhibitors treatment for 24 h. Scale bar = 200 μm. Data represent mean ± SD (*n* = 4) and are representative of two separate experiments; a = *P* < 0.05 vs DMSO, b = *P* < 0.05 vs geda 111 nm; one‐way ANOVA Fisher's test. See Table S14 for data. Alpe, alpelisib; capi, capivasertib; eve, everolimus; geda, gedatolisib; samo, samotolisib; RU, relative units.

Due to the direct and indirect involvement of the PAM pathway in cytoskeleton remodeling and cell migration, PAM inhibitors have been shown to decrease migration and invasion of cancer cells, where glycolysis at the cell periphery is essential [47, 48]. Preliminary experiments using the CELsignia PI3K Signaling Pathway test to evaluate PI3K‐related signaling pathway activity showed that gedatolisib significantly inhibited PI3K signaling after stimulation with the GPCR agonist LPA (125 nm) in Du145 cells (Fig. 6A). Since the CELsignia test measures changes in cell impedance, which can be due to changes in cell‐adhesion and cell movement, these preliminary results suggested that gedatolisib may affect PC cell migration.

To directly test the effects of PAM inhibitors on cell migration, Du145 cells were tested in the Transwell migration assay. Cells seeded in FBS‐free medium onto the top chamber were induced to migrate by using medium supplemented with FBS and LPA as chemoattractant in the lower chamber. Cells in the upper chamber were treated with PAM inhibitors and let migrate to the lower chamber for 24 h. As shown in Fig. 6B and Table S14, gedatolisib (111 nm) inhibited cell migration up to 64%, compared to 38%, 18%, 13%, and no inhibition for samotolisib (111 nm), alpelisib (1000 nm), everolimus (111 nm), and capivasertib (1000 nm) respectively. These results suggest that PC cell migration involves multiple nodes of the PAM pathway and cannot be effectively controlled when only one node is inhibited.

### 3.7 *In vivo* efficacy of gedatolisib in prostate cancer xenograft models

**Fig. 7:**
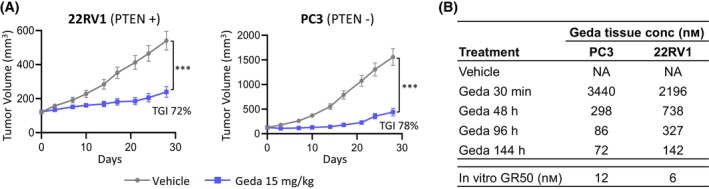
Gedatolisib *in vivo* efficacy in prostate cancer (PC) xenograft models. (A) Growth curves of 22RV1 and PC3 xenograft tumors after treatment with vehicle or gedatolisib (15 mg·kg^−1^, iv, Q4D; BALB/c nude castrated mice, 10 mice/arm). Data represent mean ± SEM. ***P < 0.001, one‐way ANOVA. The individual tumor weights are shown in Table S15 (22RV1) and Table S16 (PC3). (B) Pharmacokinetic analysis of gedatolisib concentration in xenograft tissue samples at different time points after the last gedatolisib dose (day 28). Data represent the mean of two independent tumor samples. Geda, gedatolisib; NA, not applicable.

Since gedatolisib showed the highest *in vitro* efficacy among the PAM inhibitors tested, mouse studies were conducted to test gedatolisib efficacy *in vivo*. Gedatolisib was tested in cell line‐derived mouse xenograft models of PTEN‐positive (22RV1) and PTEN‐negative (PC3) prostate cancer. As shown in Fig. 7A and Tables S15 and S16, treatment with gedatolisib (15 mg·kg^−1^) for 28 days induced significant (*P* < 0.001) tumor growth inhibition (TGI) in both 22RV1 (72%) and PC3 (78%). During the 28 days dosing period the body weight of the mice did not change significantly, indicating that gedatolisib did not have toxic effects (Fig. S4). Pharmacokinetic analysis of tumor tissue samples collected at early (30 min) and late (48, 96, 144 h) time points after the last gedatolisib dose showed that gedatolisib concentration at 96 h (i.e. the time between two doses) was 86 nm for PC3 and 327 nm for 22RV1, which was well above the *in vitro* GR_50_ for these cell lines (12 nm for PC3 and 6 nm for 22RV1) (Fig. 7B). Of note, the levels of gedatolisib in the PC xenograft models were below the gedatolisib levels detected in patients treated with 1 cycle of 154 mg gedatolisib, where gedatolisib *C*_Max_ in plasma was 9988 ng·mL^−1^ (> 15 μm) and the *T*_1/2_ was 36 h [26]. These results showed that gedatolisib effectively inhibited PAM pathway activity and tumor growth in PC models *in vivo*.

## 4 Discussion

Increased activation of the PAM pathway occurs frequently in PC and is associated with cancer progression and acquisition of castration‐resistance and androgen/AR‐independence after ADT or treatment with AR pathway inhibitors. Due to the reliance of PC cells on metabolic adaptations induced by PAM pathway activation, this pathway is an attractive target for PC therapy. However, targeting single nodes of the PAM pathway can be hampered by resistance mechanisms due to feedback loops or compensatory pathways among the PAM pathway nodes. It has been hypothesized that multi‐node PAM inhibitors should be more effective than single‐node inhibitors because they may overcome resistance mechanisms associated with a more narrowly targeted inhibition of the PAM pathway. Our study showed that equipotent multi‐node targeting of the PAM pathway by gedatolisib induced greater growth‐inhibitory and cytotoxic activity compared to single‐node PAM inhibitors in PC cells. By using a series of functional and metabolic assays, we further showed that the differential effect of single‐node versus multi‐node PAM inhibitors on cell growth can be traced to a differential effect on critical PAM‐controlled functions, including cell survival, cell cycle, cell migration, protein synthesis, oxygen consumption rate, and glycolysis.

Both GR metrics analyses and endpoint cell viability assays showed that gedatolisib generally exerted greater anti‐proliferative and cytotoxic effects compared to single‐node PAM inhibitors (alpelisib, capivasertib, everolimus) or to a less uniformly potent pan‐PI3K/mTOR inhibitor (samotolisib), regardless of the PTEN status or androgen sensitivity of the PC cells. The PAM inhibitors potency and efficacy results presented here are consistent with published *in vitro* studies showing similar IC_50_ ranges in cancer cell lines [17, 32, 39, 49, 50, 51]. Moreover, as previously described, targeting AKT with capivasertib tended to be more effective in PTEN‐negative cell lines, while targeting PI3Kα with alpelisib tended to be more effective in PTEN‐positive cell lines [15]. We also confirmed and extended a recent study reporting that the pan‐PI3K inhibitor copanlisib was more effective than AKT or PI3Kα inhibitors in androgen‐sensitive PC cell lines [52]. Our experiments showed that pan‐PI3K/mTOR inhibition by gedatolisib induced greater growth‐inhibitory effects than pan‐PI3K inhibition by copanlisib, regardless of androgen sensitivity or PTEN status. This suggests that equipotent mTOR inhibition is required in addition to PI3K inhibition to increase efficacy as well as to target a larger subpopulation of patients with PC. This hypothesis is also corroborated by the observation that samotolisib, which has a weaker activity against mTOR, was less effective than gedatolisib.

The differential effects of single‐node and multi‐node PAM inhibitors on GR metrics may be attributed to a differential effect on both cell cycle and apoptosis. The PAM pathway plays an important role in promoting cell cycle progression and cell proliferation through multiple PAM pathway effectors, including the AKT‐GSK3 axis, the AKT‐FOXO axis, and mTORC1 [3, 4]. In addition, several AKT effectors, such as BCL2‐associated agonist of cell death (BAD) and FOXO transcription factors, are inhibited by AKT, and their inhibition, directly or indirectly, prevents apoptosis and promotes cell survival [1, 3, 4]. AKT‐independent mechanisms downstream of PI3K and mTOR can also be involved in cancer cell survival and apoptosis [1]. For instance, Will et al. [40] reported that inhibition of PI3K can induce apoptosis through AKT‐independent inhibition of the RAS–ERK pathway in breast cancer cells. Interestingly, in the same study, AKT inhibition did not induce cell death. Our study showed that inhibition of both PI3K and mTOR blocked cell cycle and induced apoptosis more effectively than AKT, PI3Kα, or mTOR inhibitors.

The differential effect on apoptosis and cell death was especially apparent in PTEN‐positive cells (e.g., 22RV1). Oncogenic activation of PAM signaling was shown to activate a negative feedback loop, whereby PTEN expression is induced through 4EBP1‐mediated translational regulation [53]. In the presence of PTEN, this feedback can potentially be relieved by PAM pathway inhibition, resulting in PI3K activation. Multi‐node PAM inhibition exerted by gedatolisib may have prevented feedback relief by inhibiting both PI3K and mTOR. Our results also indicated that pan‐PI3K/mTOR inhibition exerted more durable cell death effects than single‐node PAM inhibition. Gedatolisib treatment for 24 h followed by 48 h wash out was sufficient to induce almost the same level of cell death induced by continuous treatment for 72 h. This “hit and run” effect was not observed with the single‐node PAM inhibitors, which appeared to act slower. This suggests that if single‐node PAM inhibitors are not continuously present at sufficiently high concentrations, cells may have enough time to develop adaptation mechanisms that would not develop in the response to a fast‐acting multi‐node PAM inhibitor like gedatolisib.

The efficacy of PAM inhibitors is frequently assessed by measuring markers of PAM activity such as phosphorylation of pRPS6 and p4EBP1 [54]. Under our experimental conditions, the differential effects of single‐node versus multi‐node PAM inhibitors on growth rate and cell viability correlated with inhibition of p4EBP1, while they not always correlated with inhibition of pRPS6. This was especially apparent for everolimus, which potently reduced pRPS6 in all cell lines, but exerted modest growth‐inhibitory effects in most of the cell lines tested. Sathe et al. [55] reported that to efficiently decrease p4EBP1, both PI3K and mTORC1 need to be inhibited. In addition, Lee et al. [56] showed that inhibition of 4EBP1 phosphorylation was sufficient to suppress cancer cell proliferation and was required to achieve maximal tumor growth inhibition. This could in part explain why gedatolisib (which targets PI3K and mTOR with similar potency) was more effective at inhibiting p4EBP1 and cell growth than the single‐node PAM inhibitors or a pan‐PI3K/mTOR inhibitor with weaker potency against mTOR.

In addition to pRPS6 and p4EBP1, pAKT has also been used extensively as a marker of PAM pathway activity [54]. Consistently, in addition to pRPS6 and p4EBP1, gedatolisib has been shown to inhibit both pAKT(S473) and pAKT(T308) [17]. However, in initial studies on breast cancer cell lines, we found that pAKT was a less robust marker than pRPS6 and p4EBP1 and showed a poorer correlation with viability metrics (data not shown). While AKT phosphorylation may be indicative of PAM pathway activity and PAM inhibitors response in some contexts, the presence of multiple AKT phosphorylation sites and activity states, the transient nature of AKT phosphorylation, and feedback mechanisms leading to AKT reactivation can make the interpretation of this phospho‐marker more challenging than other markers [4, 57]. In addition, pAKT inhibition is not a suitable marker for some PAM inhibitors, e.g., capivasertib, which increases pAKT [49]. To address the intrinsic limitations of using phospho‐markers to assess PAM pathway activity, we extensively analyzed PAM‐controlled functions (e.g. cell cycle, survival, protein synthesis, metabolism) that could offer a more biologically relevant readout of the response to gedatolisib or the other PAM inhibitors.

The differential effect of the PAM inhibitors on p4EBP1 was paralleled by a differential effect on protein synthesis. Upon PAM pathway activation, mTORC1 phosphorylates 4EBP1 and triggers its dissociation from eukaryotic initiation factor 4E (eIF4E), allowing cap‐dependent mRNA translation [41, 42]. eIF4E‐mediated translation initiation is a critical driver of PC development and progression [58]. Moreover, loss of AR, which normally acts as a negative regulator of translation by exerting a direct transcriptional control on 4EBP1, promotes the assembly of the translation initiation complex, leading to de‐repression of translation initiation and increased cell proliferation [59]. The control of protein translation plays a key role in cancer development and progression because proteins synthesis is required to increase cancer cells' biomass for cell division, as well as to translate proteins involved in tumor growth and invasion (e.g., BCL‐xL, cyclins, MMP3, VEGF) [41, 42]. In this respect, it is intriguing that the translation of pro‐invasive mRNAs through the 4EBP1‐eIF4E axis in PC cells is suppressed by dual mTORC1/2 inhibition and not by mTORC1 inhibition alone [60]. Our results showed that gedatolisib, by inhibiting PI3K as well as mTORC1 and mTORC2, reduced protein synthesis more effectively than single‐node PAM inhibitors. The more effective inhibition of protein synthesis could further explain the greater growth‐inhibitory and anti‐migratory effects observed in response to gedatolisib.

A relevant finding of the present study is that single‐node and multi‐node PAM inhibitors had a different impact on PC cell metabolism related to glucose and O_2_ consumption and lactate production. Cancer cells undergo extensive metabolic reprogramming (e.g. increased glucose uptake and glycolysis) to accommodate the increased energy and the anabolic demands required by cancer cells to sustain cell growth and to overcome hostile microenvironmental conditions (e.g. hypoxia, low nutrients) [46]. PC cancer cells acquire unique metabolic adaptations that mirror the unique metabolism of their normal counterpart [43, 61]. Differently from other epithelial cells, the tricarboxylic acid cycle (TCA) of prostate epithelial cells is truncated after citrate oxidation, and most of the energy production is derived from aerobic glycolysis [62, 63]. At early cancer stages, PC cells undergo a metabolic reprogramming that restores the TCA and makes cells more dependent on oxidative phosphorylation (OXPHOS) and less dependent glycolysis [64]. At advanced cancer stages, glycolysis is promoted again through increased expression of glycolytic enzymes [65, 66], and PC cells can produce energy through multiple energy source pathways [67]. In addition, the citrate produced from glucose carbon sources through glycolysis and the TCA can be used for lipid biosynthesis. *De novo* lipid production characterizes early PC stages and is exacerbated during disease progression to sustain cancer cell proliferation [68, 69].

These metabolic changes were captured by our RNA‐seq analysis of PC clinical progression showing concomitant upregulation of transcripts involved in PAM pathway signaling, hypoxia and glucose metabolism (Fig. 5A). Since PAM pathway activation is pivotal for PC metabolic reprogramming [11], PAM inhibitors can target critical metabolic vulnerabilities underlying PC progression. We demonstrate that simultaneously antagonizing PI3K and mTOR with gedatolisib is more effective at reducing glucose and oxygen consumption by PC cells than single‐node PAM inhibitors. Moreover, ongoing experiments in prostate cancer cell lines suggest that the reduced glucose consumption induced by gedatolisib could in turn translate into reduced lipid biosynthesis and storage (Khan S. et al., manuscript in preparation).

The metabolic changes induced by gedatolisib can directly impact cancer cells by reducing catabolic and anabolic activities necessary for cell growth and proliferation [11, 46]. Moreover, since glycolysis plays a relevant role in promoting EMT and preventing apoptosis in cancer cells [70, 71], glucose metabolism inhibition could contribute to the decreased migration and induction of apoptosis in response to gedatolisib. In addition, the metabolic changes caused by gedatolisib could also influence the tumor microenvironment (TME). By inducing hypoxia, decreasing pH (consequent to high lactate), and lowering glucose in the TME, cancer cells can promote immune suppressor cells while imposing metabolic restrictions to anti‐tumor immune cells [44]. The inhibition of OCR and glycolysis in cancer cells by gedatolisib could lead to a “normalization” of the TME with consequent improvement of the anti‐tumor immune response. In support of this hypothesis, Yan et al. [72] showed that gedatolisib can induce anti‐tumor immune cell infiltration and activation in the PyMT mouse model of mammary carcinoma.

The growth‐inhibitory effect of pan‐PI3K/mTOR inhibition in different PC cell contexts was further confirmed *in vivo*. The PC xenograft studies presented here showed that gedatolisib induced > 70% TGI in two cell line models, one PTEN‐positive (22RV1) and one PTEN‐negative (PC3), with described resistance to AR inhibitors [73]. Based on our *in vitro* data, as well as published evidence that gedatolisib induced apoptosis in breast and ovarian cancer xenograft models [17, 18, 74], it is plausible that the TGI observed in the PC xenograft models is due to a combination of cell cycle inhibition and induction of cell death. Although we have not compared the effect of gedatolisib with single‐node PAM inhibitors *in vivo*, previously published studies have shown that capivasertib (100–150 mg·kg^−1^, BID) induced no or very modest TGI in both 22RV1 and PC3 xenograft models [75, 76, 77]; similarly everolimus (5 mg·kg^−1^ twice a week) inhibited tumor growth < 30% in the PC3 xenograft model [78]. These observations suggest that single node inhibition of AKT or mTOR is less efficacious than pan‐PI3K/mTOR inhibition *in vivo*.

Hyperglycemia is a relevant adverse effect frequently observed in patients treated with PAM inhibitors [79]. Clinical studies [24, 26] have shown reduced rate of hyperglycemia in patients treated with gedatolisib relative to published data for single‐node PAM inhibitors like alpelisib and everolimus [20, 21]. The link between PAM inhibitors and hyperglycemia has been largely based on studies employing approved drugs or compounds that target single protein types in the PAM signaling pathway. The outcomes from these studies may have been more strongly affected by the route of administration leading to high concentration exposure in the liver before systemic distribution, pharmacokinetic/pharmacodynamic properties, target affinity, cellular location or sequestration of the compound. However, we cannot rule out that the mechanism of action on different cell types (e.g. hepatic, pancreatic, tumor, muscles) of a pan‐PI3K/mTOR inhibitor leads to fundamentally different outcomes with respect to the effect on biochemical carbon homeostasis when compared to single‐node PAM inhibitors. The effect of various PAM inhibitors on glucose metabolism in cancer cells has been well described [79]. However, the complexity of systemic PAM inhibition includes effects on glucagon and insulin release. Besides glucose, both of these hormones are regulated by key metabolic carbon sources including fatty acids and amino acids, both of which affect PAM [80]. Since gedatolisib appears to decrease glucose consumption and glycolysis to a greater extent than single‐node PAM inhibitors, a potential reduction in glucagon/insulin response could be due to decreased gluconeogenesis and/or glycogenolysis [79]. Currently, there is no clear biochemical explanation for the significant differences in hyperglycemia for single‐node inhibitors versus gedatolisib. The answer will most likely be found in additional functional studies employing the unique properties of gedatolisib for the release of insulin and glucagon by α and β islet cells and the impact on metabolic function of muscle and hepatic cells.

Several pan‐PI3K/mTOR inhibitors have been developed, but clinical results have been disappointing mostly due to dose‐limiting toxicities, limited efficacy, or drug instability. For instance, samotolisib has been tested in combination with enzalutamide in patients with mCRPC in a recent Phase 1b/2 trial and showed tolerable side effects, clinical benefit in PTEN+ patients but not PTEN‐negative patients, and metabolic instability [81]. Based on our *in vitro* data, samotolisib's limited efficacy in PTEN‐negative patients may be in part due to its relatively low potency against mTOR. Gedatolisib is well‐tolerated in patients and is equally potent against Class I PI3K and mTOR. Preliminary results from the first‐in‐human gedatolisib study and from a phase 1b clinical trial evaluating gedatolisib combined with palbociclib and endocrine therapy in advanced breast cancer showed promising efficacy with relatively few adverse effects [24, 26]. Based on these encouraging results, a Phase 3 clinical trial (VIKTORIA‐1, NCT05501886) is currently evaluating gedatolisib plus fulvestrant, with and without palbociclib, in patients with advanced or metastatic breast cancer who progressed after CDK4/6 and an aromatase inhibitor therapy. More recently, a Phase 1/2 clinical trial (CELC‐G‐201, NCT06190899) has been initiated to test gedatolisib in combination with darolutamide in patients with mCRPC previously treated with an AR inhibitor.

## 5 Conclusions

The increased activation of the PAM pathway in PC and its connection with resistance mechanisms to androgen‐targeted therapy has led to several non‐clinical and clinical studies evaluating PAM inhibitors, especially in combination with AR pathway inhibitors. Our study underscores the relevance of targeting multiple nodes, versus single nodes, of the PAM pathway to maximize the inhibition of critical PAM‐controlled functions and to achieve effective anti‐proliferative and cytotoxic effects in PC cells, regardless of PTEN status or androgen sensitivity.

## Conflict of interest

AS, SK, SR, AB, IM, AD, JM, LD, CI, MS, RK, SS and LL are all employed by and/or have ownership interest in Celcuity, Inc.

## Author contributions

AS contributed to the study design, experiments execution and data analyses, bioinformatics, and drafted the manuscript figures; SK contributed to the study design, performed experiments and data analyses, and contributed to the manuscript draft; SR contributed to the data analysis, critical thinking, and drafting and revising the manuscript; AB contributed to assays' optimization; IM contributed to the study design and animal studies; AD contributed to the animal studies; JM contributed to cell culture maintenance; LD, CI and MS provided technical support; RK and SS optimized the metabolic assays; LL contributed to the study design, and drafting, revising, and finalizing the manuscript.

### Peer review

The peer review history for this article is available at https://www.webofscience.com/api/gateway/wos/peer‐review/10.1002/1878‐0261.13703.

## Supporting information


**Fig. S1.** Analysis of PC cell viability inhibition by PAM inhibitors.
**Fig. S2.** Analysis of additional PAM inhibitors response in PC cell lines using GR metrics.
**Fig. S3.** Analysis of glucose uptake in response to PAM inhibitors.
**Fig. S4.** Mouse body weight in PC3 and 33RV1 xenografts treated with vehicle or gedatolisib.

**Table S1.** GR values in PC cell lines treated with PAM inhibitors for 72 h in medium plus charcoal‐stripped FBS and 1 nm DHT.
**Table S2.** Cell viability (% inhibition) in PC cell lines treated with PAM inhibitors for 72 h.
**Table S3.** GR values in PC cell lines treated with PAM inhibitors for 72 h.
**Table S4.** Cell Cycle analysis in PC cell lines treated with PAM inhibitors for 48 h.
**Table S5.** EdU incorporation (% inhibition) in PC cell lines treated with PAM inhibitors for 48 h.
**Table S6.** Cell death and apoptosis in PC cell lines treated with PAM inhibitors for 48 h.
**Table S7.** Cell death induction in PC cells treated with PAM inhibitors for 48 h.
**Table S8.** p4EBP1 inhibition in PC cells treated with PAM inhibitors for 48 h.
**Table S9.** Inhibition of OPP incorporation, pRPS6, and p4EBP1 in PC cells treated with PAM inhibitors for 24 h.
**Table S10.** List of genes used in temporal transcript‐mapping plots.
**Table S11.** Lactate produced in PC cell lines treated with PAM inhibitors for 24 h.
**Table S12.** Glucose uptake in PC cells treated with PAM inhibitors for 4 h.
**Table S13.** OCR in PC cell lines treated with PAM inhibitors for 24 h.
**Table S14.** Du145 Cell migration after 24 h treatment with PAM inhibitors.
**Table S15.** Tumor volume in individual 22RV1 xenograft mice treated with vehicle or gedatolisib.
**Table S16.** Tumor volume in individual PC3 xenograft mice treated with vehicle or gedatolisib.

## Data Availability

All data are available in the main text or the supplementary materials. The dataset analyzed during the current study are available from the corresponding author upon reasonable request.
