# Supplementary material for: Assessments of prostate cancer cell functions highlight differences between a pan‐PI3K/mTOR inhibitor, gedatolisib, and single‐node inhibitors of the PI3K/AKT/mTOR pathway
Source: Mol Oncol. 2024 Aug 2;19(1):225–47. doi: 10.1002/1878-0261.13703 (PMC11705819; doi:10.1002/1878-0261.13703)
Supplement: Supplementary file 1 — Fig. S1. Analysis of PC cell viability inhibition by PAM inhibitors. Fig. S2. Analysis of additional PAM inhibitors response in PC cell lines using GR metrics. Fig. S3. Analysis of glucose uptake in response to PAM inhibitors. Fig. S4. Mouse body weight in PC3 and 33RV1 xenografts treated with vehicle or gedatolisib. [file MOL2-19-225-s001.docx]

**Supplementary Information**

**Assessments of prostate cancer cell functions highlight differences between a pan-PI3K/mTOR inhibitor, gedatolisib, and single-node inhibitors of the PI3K/AKT/mTOR pathway**

Adrish Sen, Salmaan Khan, Stefano Rossetti, Aaron Broege, Ian MacNeil, Ann DeLaForest, Jhomary Molden, Laura Davis, Charles Iversrud, Megan Seibel, Ross Kopher, Stephen Schulz, Lance Laing*

Celcuity, Inc. 16305 36^th^ Ave N, Suite 100, Minneapolis, MN 55446

***Corresponding Author**

**Email:** [llaing@celcuity.com](mailto:llaing@celcuity.com)

<https://orcid.org/0000-0003-4593-0685>


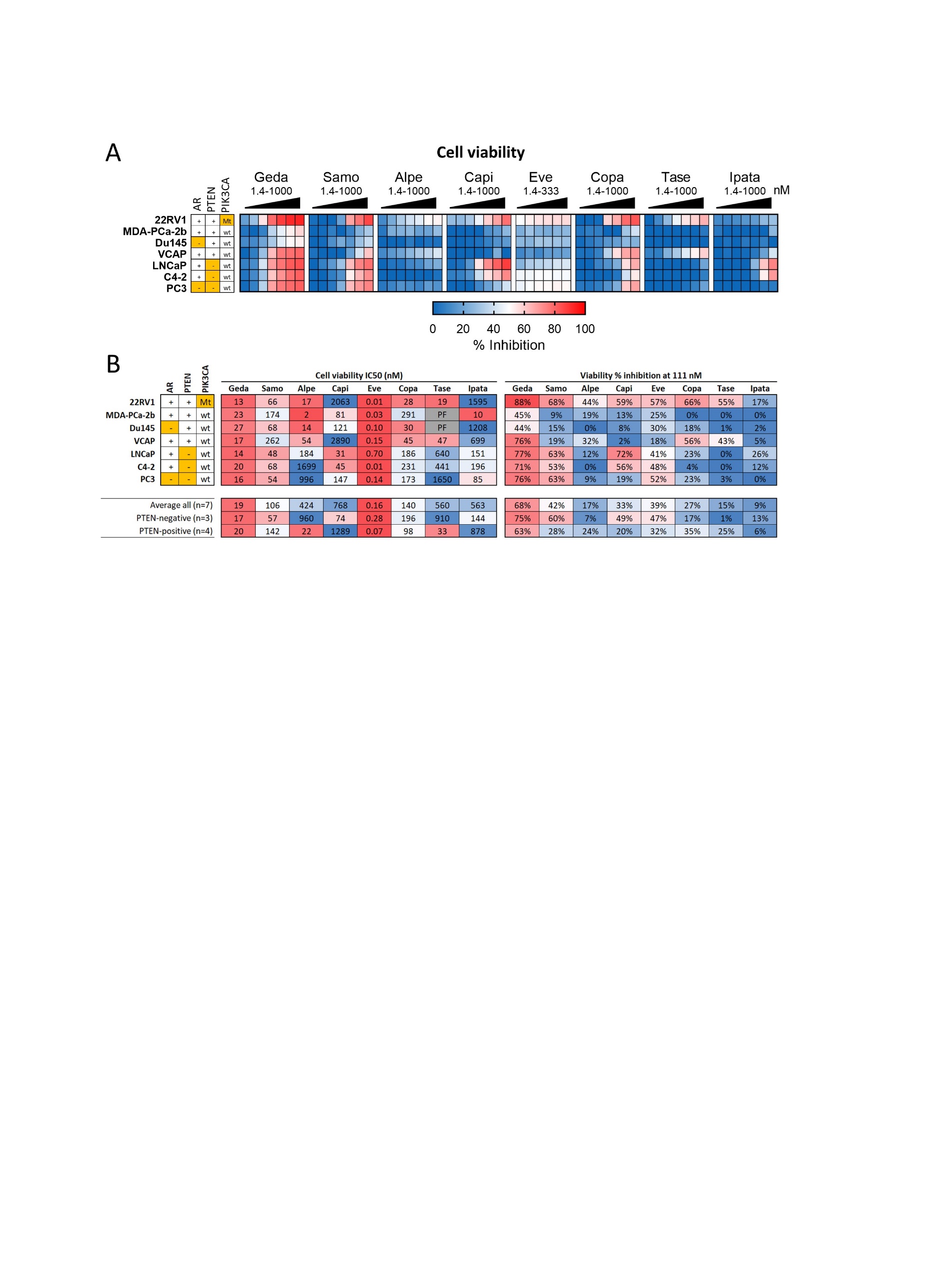


**Figure S1.** Analysis of PC cell viability inhibition by PAM inhibitors. **A.** Heatmap showing inhibition of cell viability (assessed by RTGlo MT assay) after treatment with increasing concentration of PAM inhibitors for 72 hours in 7 PC cell lines. % inhibition is relative to control cells treated with DMSO. See Table S2 for data. **B.** Cell viability absolute IC_50_ values show that gedatolisib is more potent than the other PAM inhibitors tested; the % inhibition of cell viability shows that gedatolisib is more efficacious than the other PAM inhibitors at 111 nM. Average IC_50_s in PTEN+ or PTEN- subpopulations are shown. PF = poor fit prevented reliable IC_50_ calculation.


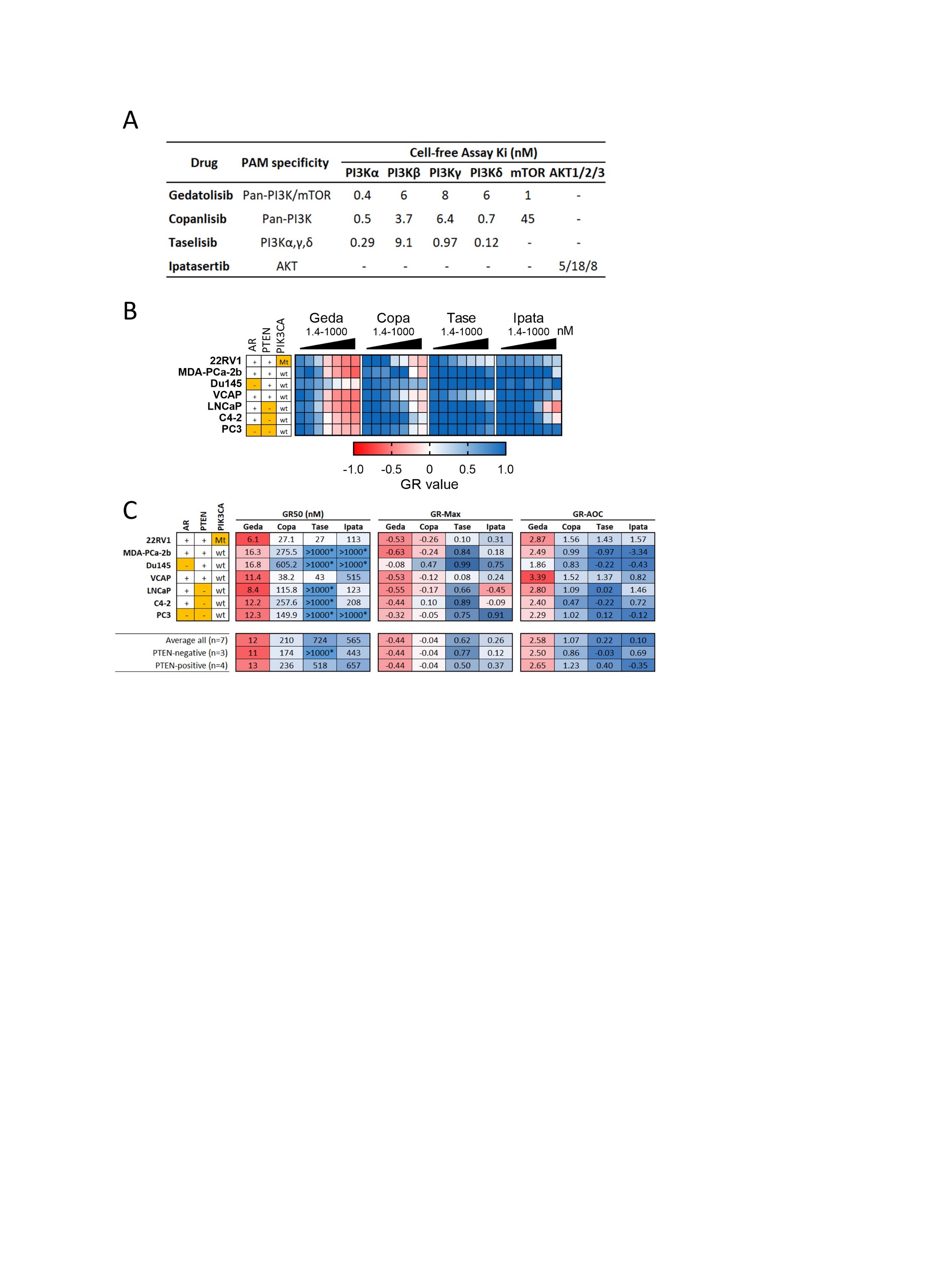


**Figure S2.** Analysis of additional PAM inhibitors response in PC cell lines using GR metrics. **A.** Specificity and affinity of gedatolisib compared to copanlisib, taselisib, and ipatasertib. **B**. Heatmap showing GR values in 7 PC cell lines treated for 72 hours with increasing PAM inhibitors concentrations. See Table S3 for data. **C.** Summary of PAM inhibitors GR_50_, GR_Max_ and GR_AOC_ in the PC cell lines tested. Average values in PTEN-positive and PTEN-negative subpopulations are shown. * = Max concentration tested, GR_50_ not reached.


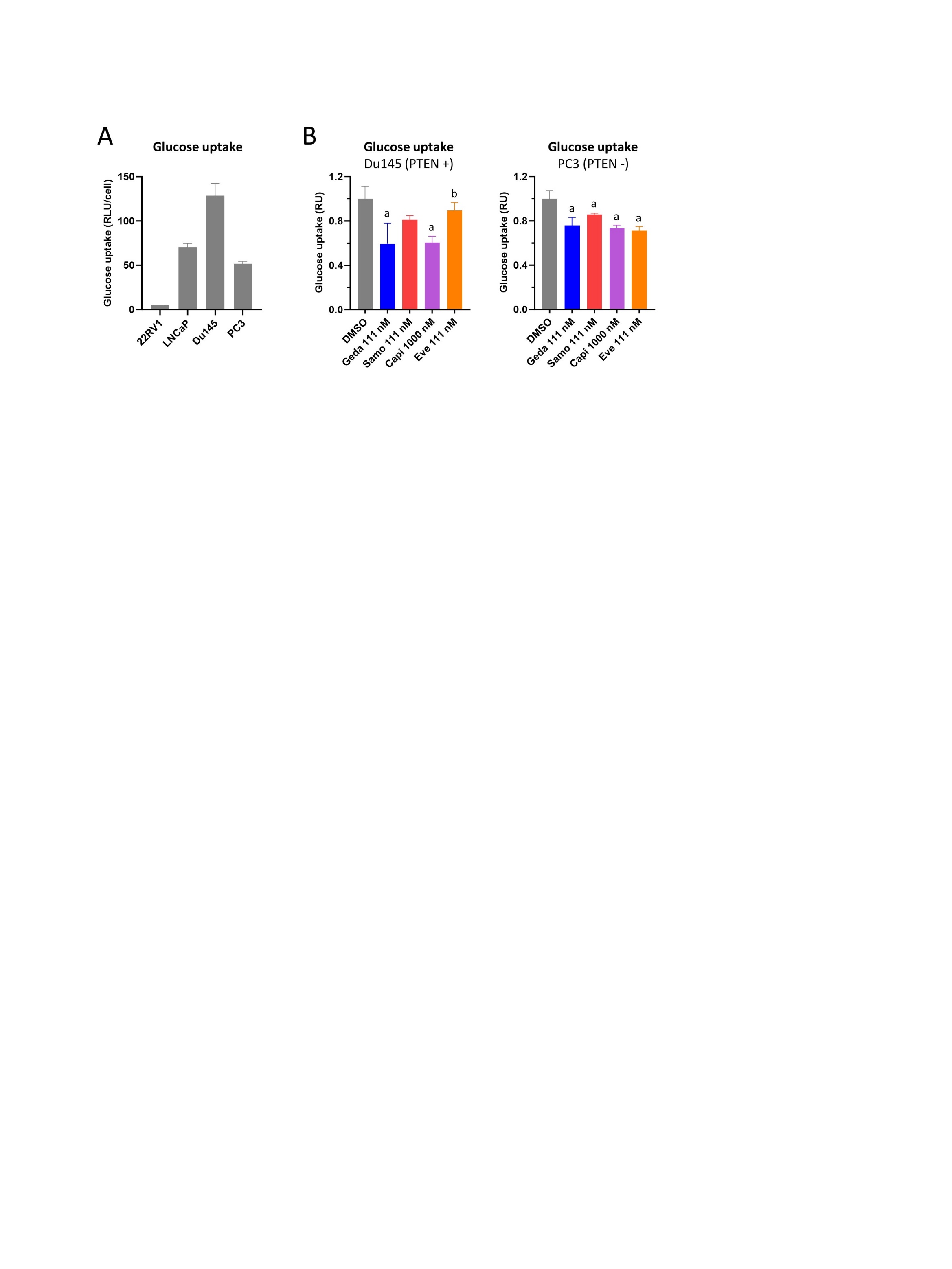


**Figure S3.** Analysis of glucose uptake in response to PAM inhibitors. **A.** Baseline glucose uptake in PC cell lines assessed by Glucose uptake Glo assay. Luminescence was normalized to cell number; data represent mean ± SD (n = 2-4). **B.** Glucose uptake analysis in Du145 and PC3 cells treated with PAM inhibitors for 4 hours. Glucose uptake was normalized to cell number (assessed by RT-Glo MT assay). Data represent mean ± SD (n = 3); a = p < 0.05 vs DMSO, b = p < 0.05 vs 111 nM gedatolisib; one-way ANOVA Fisher’s test. See Table S12 for data.


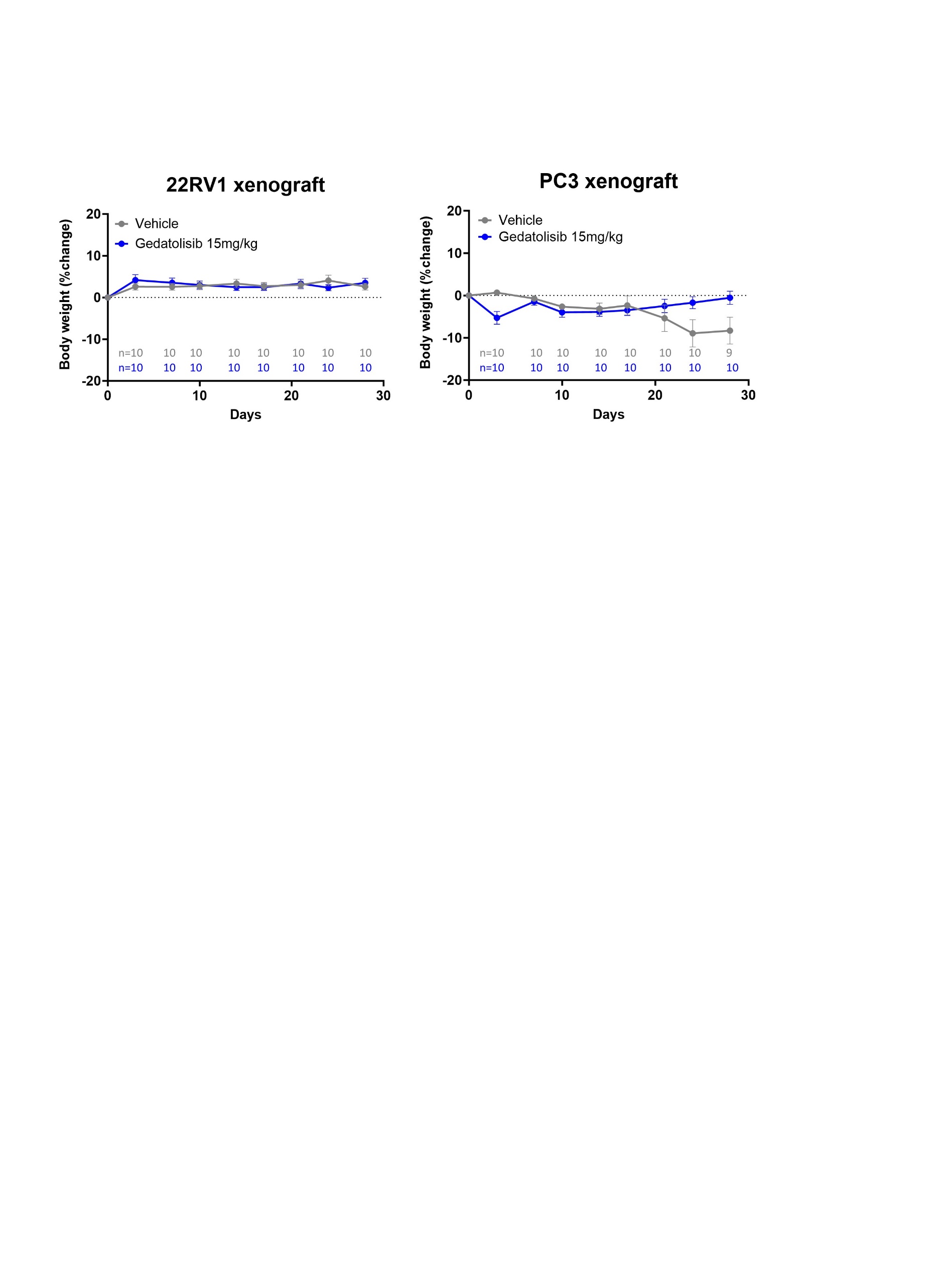


**Figure S4.** Mouse body weight in PC3 and 33RV1 xenografts treated with vehicle or gedatolisib for 28 days. n = number of mice.
